# Tandem Duplications and the Limits of Natural Selection in *Drosophila yakuba* and *Drosophila simulans*

**DOI:** 10.1371/journal.pone.0132184

**Published:** 2015-07-15

**Authors:** Rebekah L. Rogers, Julie M. Cridland, Ling Shao, Tina T. Hu, Peter Andolfatto, Kevin R. Thornton

**Affiliations:** 1 Ecology and Evolutionary Biology, University of California, Berkeley, California, United States of America; 2 Ecology and Evolutionary Biology, University of California, Davis, Davis, California, United States of America; 3 Ecology and Evolutionary Biology, University of California, Irvine, Irvine, California, United States of America; 4 Ecology and Evolutionary Biology and the Lewis Sigler Institute for Integrative Genomics, Princeton University, Princeton, New Jersey, United States of America; University of Iceland, ICELAND

## Abstract

Tandem duplications are an essential source of genetic novelty, and their variation in natural populations is expected to influence adaptive walks. Here, we describe evolutionary impacts of recently-derived, segregating tandem duplications in *Drosophila yakuba* and *Drosophila simulans*. We observe an excess of duplicated genes involved in defense against pathogens, insecticide resistance, chorion development, cuticular peptides, and lipases or endopeptidases associated with the accessory glands across both species. The observed agreement is greater than expectations on chance alone, suggesting large amounts of convergence across functional categories. We document evidence of widespread selection on the *D. simulans* X, suggesting adaptation through duplication is common on the X. Despite the evidence for positive selection, duplicates display an excess of low frequency variants consistent with largely detrimental impacts, limiting the variation that can effectively facilitate adaptation. Standing variation for tandem duplications spans less than 25% of the genome in *D. yakuba* and *D. simulans*, indicating that evolution will be strictly limited by mutation, even in organisms with large population sizes. Effective whole gene duplication rates are low at 1.17 × 10^−9^ per gene per generation in *D. yakuba* and 6.03 × 10^−10^ per gene per generation in *D. simulans*, suggesting long wait times for new mutations on the order of thousands of years for the establishment of sweeps. Hence, in cases where adaptation depends on individual tandem duplications, evolution will be severely limited by mutation. We observe low levels of parallel recruitment of the same duplicated gene in different species, suggesting that the span of standing variation will define evolutionary outcomes in spite of convergence across gene ontologies consistent with rapidly evolving phenotypes.

## Introduction

Tandem duplications are an essential source of genetic novelty that is useful for the development of novel traits [1–3] and their prevalence in populations is therefore expected to influence the arc of evolutionary trajectories. The observed landscape of tandem duplications in *Drosophila* spans only a few percent of the genome [4–7], and it is unclear to what extent duplications among new mutations or standing variation can provide a sufficient source of adaptive genetic variation. Tandem duplications produce a variety of novel gene structures including chimeric genes, recruited non-coding sequence, dual promoter genes, and whole gene duplications [4, 8, 9]. Surveys based on single sequenced reference genomes have suggested that whole gene duplications may form at low rates in comparison with SNPs, with even lower mutation rates for complex variants such as chimeric genes [4, 8, 10, 11]. Yet, these alternative genetic structures are known forces of evolutionary innovation [12–16]. Whole gene duplications often develop novel functions or specialize in ancestral functions [1], and chimeric genes are more likely still to produce novel molecular effects and play a role in adaptive evolution [12]. Although these variants contribute substantially to the evolution of genome content [8, 10, 17], their lower rates of formation may render evolution of tandem duplications more likely to be limited by mutation.

If population-level mutation rates are sufficiently large, new mutations will accumulate quickly and adaptation is expected to proceed rapidly [18]. However, if population-level mutation rates are low, then there will be long waiting times until the next new mutation and evolutionary trajectories are likely to stall at suboptimal solutions during the mutational lag [18–20]. *Drosophila* have large population sizes in comparison to other multicellular eukaryotes with *N*_*e*_ ≈ 10^5^−10^6^ [21–23] and absolute numbers of individuals sufficient to provide large numbers of SNPs at many sites every generation [24]. However, the ability of SNPs to traverse adaptive landscapes is often limited [25] and, the prevalence of other types of mutations beyond SNPs has not been systematically surveyed. Alternative genetic constructs such as chimeric genes can readily traverse mutational landscapes to obtain structures that cannot be readily reached via point mutations [26, 27] whereas whole gene duplications often free sequences from functional constraints to allow for the development of new gene functions [1, 2]. If the supply of tandem duplications is limited by mutation, we expect to see suboptimal outcomes in adaptive walks, limited ability to adapt to changing environments, and low rates of evolution through parallel recruitment of the same genetic solutions in different species.

The *Drosophila* offer an excellent model system for population genomics, allowing for a whole genome survey of the genetic landscape of standing variation across species in natural populations and determination of genetic convergence across taxa. There are multiple sequenced reference genomes for *Drosophila*, and genomes are small and compact, making whole genome population surveys using next generation sequencing readily tractable. Here, we focus on *D. yakuba* and *D. simulans*, which are separated by 12 MY of divergence [28], offering distantly related groups which are not expected to share polymorphic variation due to ancestry. Thus, we can measure the limits of standing variation and the incidence of parallel duplication across species, which should be broadly applicable to multicellular eukaryotic evolution.

Convergent evolution is regarded as the ultimate signal of natural selection: if the same solution is favored for a given environment then selection should result in similar phenotypes [29]. There are many known cases of convergent phenotypic evolution, but the understanding of convergence at the genetic level is limited to a small number of case studies across diverse clades [30]. These case studies have revealed convergent evolution through different genetic solutions in vertebrates [31–33], and arthropods [34–37]. Parallel evolution through similar genetic solutions, however, appears to be more common at mutational hotspots where high mutation rates at targeted sites produce mutations at a steady rate [38–40]. Beyond these results from natural populations, convergence has often been observed in experimental evolution and is considered a signal of selection favoring alleles [38, 40–43]. However, most studies of laboratory evolution take advantage of microbes or viruses with large population sizes roughly 10^9^−10^10^ such that every mutation is likely to be sampled every generation [38, 40] or from small populations that share a common pool of standing variation [41, 44] and may therefore be qualitatively different outcomes in comparison to natural evolution in multicellular eukaryotes. Indeed, known examples of evolution through parallel recruitment of the same genetic solutions in natural populations often occur through a common ancestral genetic pool [45] or through introgression [46]. In *D. melanogaster*, parallel selection on standing variation results in high levels of convergence at the genetic level due to shared pools of ancestral variation [47]. These results suggest that given the same mutational spectrum with which to work, convergent evolution will be common. However, whether similar genetic solutions can arise independently and result in sweeps on similar variants without shared ancestry is largely unknown.

Identifying factors that influence convergent evolution across distantly related taxa that do not share population level variation due to ancestry is essential to understanding the ways mutation limits evolution, the role of standing variation in evolutionary trajectories, and the genetic architecture of adaptation. Here, we survey standing variation for tandem duplications in *Drosophila yakuba* and *Drosophila simulans* and the role that this standing variation plays in adaptive evolution in natural populations. We identify signals of reduced diversity surrounding tandem duplications, and an overabundance of high frequency variants on the *D. simulans* X chromosome, pointing to a role for adaptation through gene duplication. We observe high levels of convergence at the level of gene ontology but limited shared variation across species at specific genes, pointing to limited rates of convergent evolution at the level of single genes. We show that the span of tandem duplications in populations is limited to a small fraction of the genome and that low mutation rates will lead to long waiting times for sweeps on new mutations. These results imply that evolution by tandem duplication will be limited by mutation and that parallel recruitment of gene duplicates across species is likely to be exceedingly rare even in the face of strong selection on similar phenotypes in different species.

## Results

We previously identified hundreds to thousands of segregating duplications in natural populations of *D. yakuba* and *D. simulans*, including large numbers of gene duplications [4]. We assess the numbers and types of gene duplications, differences in duplication rates across species and explore the limits of the landscape of standing variation for tandem duplications present in each species to determine the extent to which these variants can serve as a source of genetic novelty. Recently derived, segregating tandem duplications were previously detected using paired-end read mapping and coverage changes in *D. yakuba* and *D. simulans* in samples of 20 isofemale lines derived from natural populations of each species [4]. Using divergently oriented paired-end reads, we identified 1415 segregating tandem duplications in *D. yakuba*, in comparison to 975 in *D. simulans*. Strains have been sequenced to high coverage of 50–150X and duplicate identification methods have a 96% validation rate using PacBio long molecule sequencing, and a low false negative rate less than 1% based on comparisons across strains [4]. Thus, this dataset represents a high quality portrait of variation for population genomics. Here, we describe signals of selection acting on these tandem duplications, the limits of standing variation for tandem duplicates, and their role in adaptive evolution.

### Widespread selection on the *D. simulans* X chromosome

**Fig 1:**
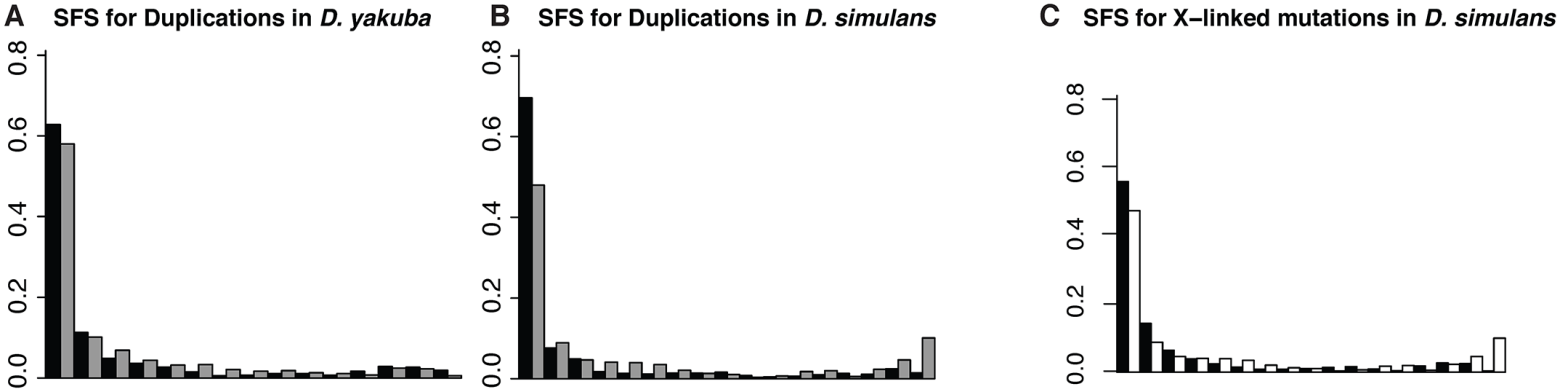
SFS for tandem duplications in *D. yakuba* and *D. simulans*, corrected for ascertainment bias. A. Site frequency spectra on the autosomes (black) and on the X (grey) in *D. yakuba*. B. SFS on the autosomes (black) and on the X (grey) in *D. simulans*. C. SFS for X-linked intronic SNPs (black) and duplicates (white) in *D. simulans*. The excess of high frequency variants on the X in *D. simulans* suggests widespread selection for tandem duplicates on the *D. simulans* X.

If tandem duplications are common targets for adaptation and selective sweeps, we should observe a shift in the site frequency spectrum (SFS) toward high frequency variants relative to neutral markers [48]. We compare the SFS for duplications with the SFS for SNPs from 8–30 bp of short introns used as a putatively neutral proxy to determine whether duplicates are subject to selection (Figure A in S1 File). The SFS for duplications is significantly different from that of intronic SNPs on the *D. simulans* autosomes using a Wilcoxon rank sum test (*W* = 268, *P* = 2.981 × 10^−6^) and *D. yakuba* autosomes (*W* = 212, *P* = 3.507 × 10^−6^). In *D. yakuba* the SFS for duplicates on the X is significantly different from that of SNPs (*W* = 211, *P* = 4.781 × 10^−4^). Duplicates show an excess of singleton variants on the autosomes in both species (Figure A in S1 File), suggesting deleterious impacts on average. We find a significant difference between the SFS of duplicates on the X chromosome and the autosomes in *D. yakuba* (*W* = 172, *P* = 0.0128) but not in *D. simulans* (*W* = 183.5, *P* = 0.1848) (Fig 1, Tables A-B in S1 File).

**Fig 2:**
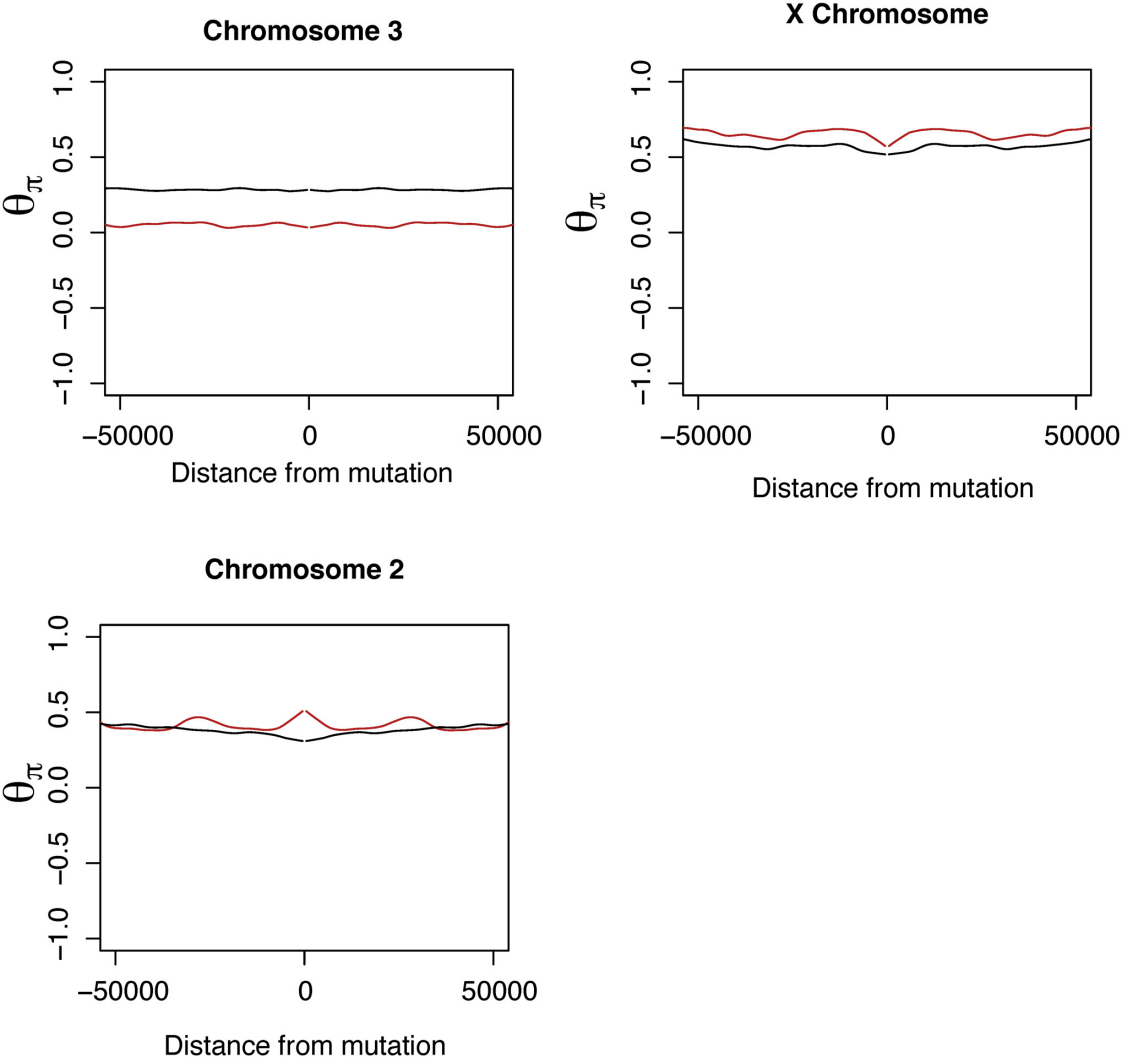
Diversity (*θ*_*π*_) as a function of distance from new mutations in *D. yakuba* for putatively neutral intronic SNPs (black) and for tandem duplications (red) by chromosome with lowess smoothing. Duplicates show a reduction in diversity approaching duplications on chromosome 3L, whereas neutral SNPs show no reduction in diversity. Plots exclude centromeric regions and the 4th chromosome which have atypical nucleotide diversity. *D. yakuba* chromosome 2 displays an atypical pattern of increased diversity and was handled separately from chromosome 3 due to segregating inversions in populations.

We have calculated average heterozygosity per site (*θ*_*π*_) [49], Wattersons’s *θ* ($\frac{S}{a}$ per site) [50], and Tajima’s *D* [51] for the four major autosomal arms and the X chromosome in *D. yakuba* and *D. simulans* using 5 kb windows with a 500 bp slide correcting for the number of sites with coverage sufficient to confidently identify SNPs (Figures B-K in S1 File). We compare *θ*_*π*_ in windows immediately surrounding tandem duplications and for windows surrounding putatively neutral SNPs from 8–30 bp of short introns to search for signals of reduced diversity consistent with selection acting on tandem duplications. These tandem duplications are polymorphic and represent putative sweeps in progress and such comparisons to within-genome controls of neutral SNPs offer greater power than alternative tests of selection [52]. A significant excess of diversity surrounding duplicates is seen on chromosome 2 in *D. yakuba* (*W* = 31594, *P* = 2.517 × 10^−4^), a putative product of alternative evolutionary dynamics driven by segregating inversions on 2L [53, 54]. We find a reduction in *θ*_*π*_ per site surrounding newly arisen tandem duplications on *D. yakuba* chromosome 3, which is not known to contain inversions (single tailed Wilcoxon rank sum test, *W* = 170168 *P* = 0.00665, see Table C in S1 File, Fig 2).

**Fig 3:**
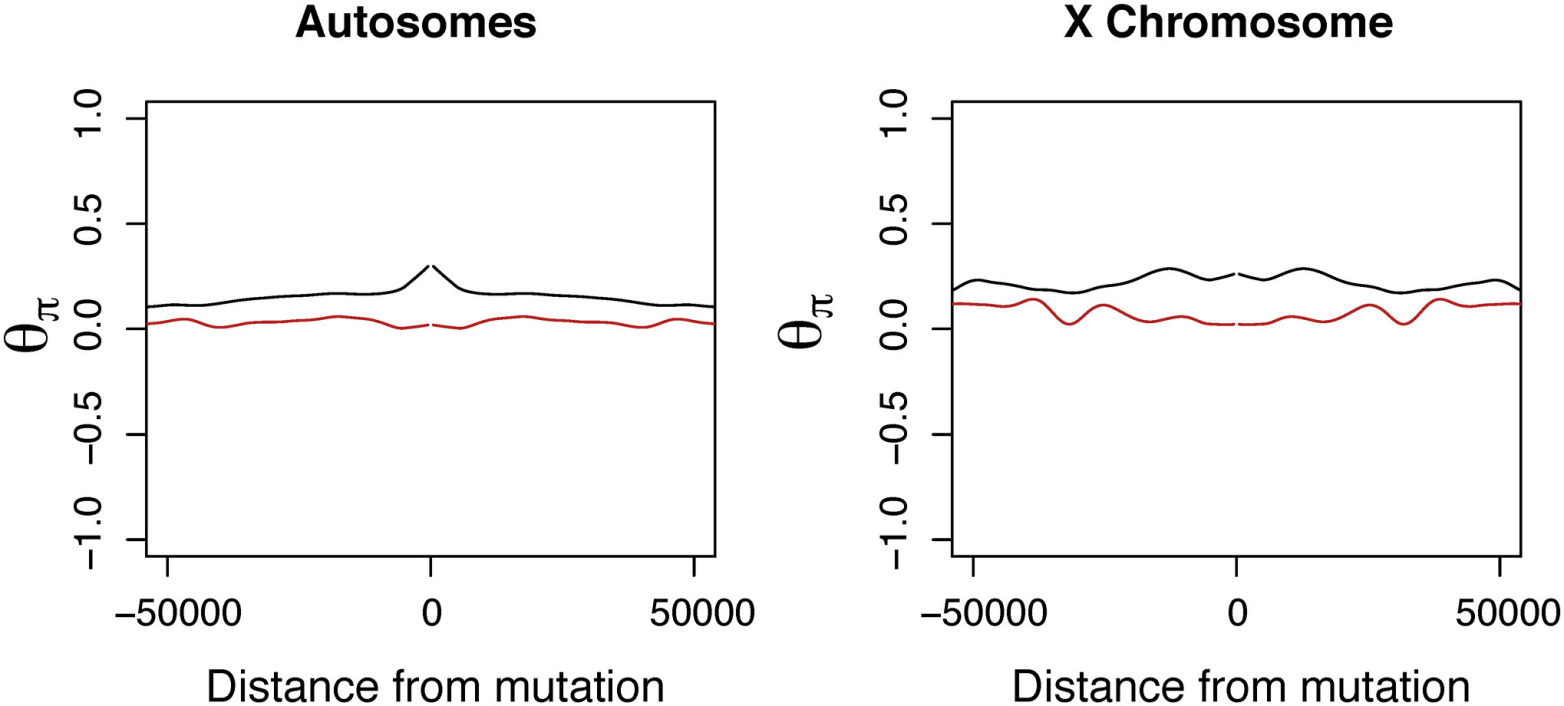
Nucleotide diversity, *θ*_*π*_ as a function of distance from new mutations in *D. simulans* for putatively neutral intronic SNPs (black) and for tandem duplications (red) by chromosome with lowess smoothing. Duplicates show a reduction in mean diversity approaching duplications on the *D. simulans* autosomes and X chromosome, whereas neutral SNPs show no reduction in diversity. Plots exclude centromeric regions and the 4th chromosome which have atypical nucleotide diversity. Chromosome 3L is strongly affected by a cluster of duplications at roughly 8.5Mb, which is excluded from the plot, but the effect is still significant without this region.

In *D. simulans* autosomes we observe a significant reduction in diversity for 5 kb windows immediately surrounding tandem duplications (single tailed Wilcoxon rank sum test, *W* = 627683.5 *P* = 2.267 × 10^−7^). Chromosome 3L in *D. simulans* contains a region encompassing multiple duplications with signals of a broad selective sweep encompassing multiple loci located at roughly 8.5 Mb that is excluded from these tests of selection (Figure I in S1 File) but results remain significant. The *D. simulans* X shows signals of reduced diversity surrounding tandem duplications (single tailed Wilcoxon rank sum test, *W* = 13450.5, *P* = 0.01819); see Table C in S1 File) (Fig 3). We observe an excess of tandem duplications at a sample frequency of 20 out of 20 sample strains (with no indication of duplication or misassembly in the resequenced reference) on the X chromosome of *D. simulans* in comparison to neutral SNPs (*P* < 10^−6^). We observe no duplicates at a sample frequency of 20 out of 20 in *D. yakuba* on the X or autosomes. Furthermore comparisons of the SFS for neutral SNPs and duplications segregating in populations show an excess of highest frequency duplicates ≥ 16 out of 17 on the *D. simulans* X (*χ* ^2^ = 21.8334, *df* = 1, *P* = 2.974 × 10^−6^). The excess of high frequency duplicates on the *D. simulans* X chromosome is indicative of selection favoring large numbers of tandem duplicates. These results imply that adaptation through duplication is common on the *D. simulans* X.

**Fig 4:**
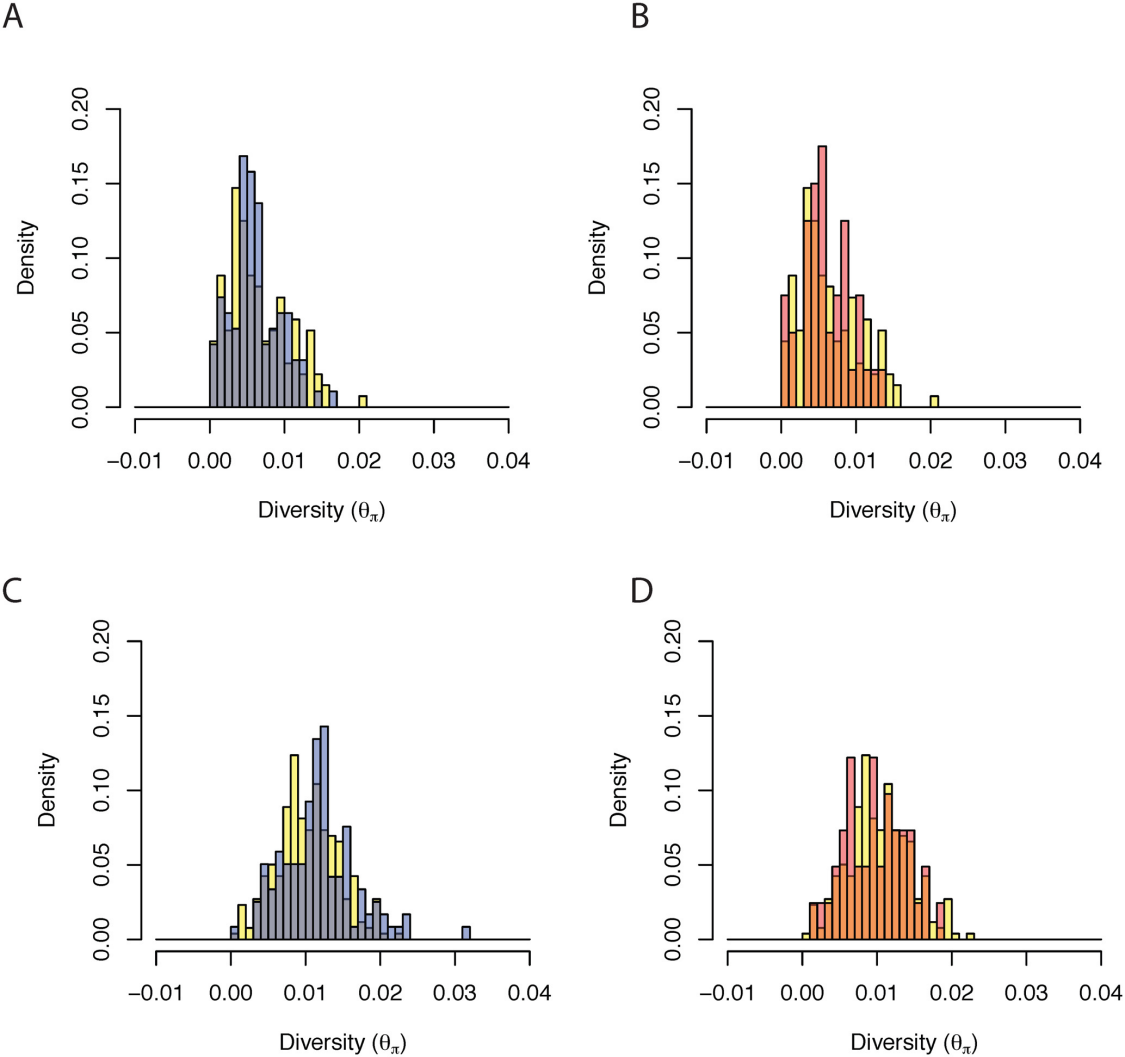
Histogram of nucleotide diversity, *θ*_*π*_, (A) For Intergenic mutations (yellow) and duplications that capture gene sequences but do not create chimeric constructs (blue) in *D. yakuba*. (B) For Intergenic mutations (yellow) and duplications that create chimeric genes (red) in *D. yakuba*. (C) For Intergenic mutations (yellow) and duplications that capture gene sequences but do not create chimeric constructs (blue) in *D. simulans*. (D) For Intergenic mutations (yellow) and duplications that create chimeric genes (red) in *D. simulans*.

It is possible that tandem duplications whose breakpoints lie within gene sequences may have different phenotypic impacts from tandem duplications that capture whole genes and do not interrupt or otherwise modify gene sequences. We compare *θ*_*π*_ for windows centered around tandem duplications that capture solely intergenic sequence with those that capture whole genes and do not create chimeric constructs with those that create chimeric genes or recruit non-coding sequence. In *D. simulans*, tandem gene duplications that do not create chimeric genes have reduced diversity (*W* = 17880, *P* = 0.0123) in comparison to mutations that capture intergenic mutations (Fig 4C–4D). Such results are consistent with selection driving an excess of whole gene duplications in *D. simulans* [4]. However, tandem duplications whose breakpoints lie within gene sequences thereby forming chimeric genes do not show similar overabundance (*W* = 5020, *P* = 0.5755). Relationships in *D. yakuba* are not significant for chimeric gene mutations (*W* = 2656, *P* = 0.8226) or whole gene duplications that do not create chimeric genes (*W* = 6387, *P* = 0.8847). Based on a binomial test, we observe a marginally significant overrepresentation of tandem duplications that capture gene sequences vs. solely non-coding sequences in *D. yakuba* (*P* = 0.0291) and highly significant for *D. simulans* (*P* = 9.044 × 10^−5^).

Populations of both *D. yakuba* and *D. simulans* show negatively skewed Tajima’s *D* for neutral SNPs, suggesting recent population expansion in both species (Figures B-K in S1 File), and similar results have been identified in *D. melanogaster* [55]. While demography and neutral evolutionary forces can result in shifts of diversity and site frequency spectra, these forces should affect sequences across individual chromosome arms and act similarly on intronic SNPs. Hence, demography is unlikely to explain the observed differences between duplicates and intronic SNPs. Further, gene conversion might putatively alter the SFS, while divergence of paralogs would artificially increase observed diversity for regions immediately surrounding tandem duplications. These forces would artificially skew statistics away from selection, leading to underreporting of the adaptive impacts of chimeric genes. Thus, we would expect many of the high frequency variants reported here to be strong candidates for ongoing selective sweeps. We further observe large numbers of singleton variants among tandem duplicates in comparison with intronic SNPs in *D. yakuba* and *D. simulans* autosomes. Copy number variants are subject to purifying selection in *D. melanogaster* [5, 56], and we observe large numbers of singleton variants in excess of neutral expectations, indicating negative selection preventing variants from rising to higher frequency. Hence, while some variants are likely to offer a means of adaptive change, many are likely to ultimately be lost from the pool of standing variation. We suggest that tandem duplications are likely to confer phenotypic impacts that are on average large enough to surpass the threshold of nearly neutral effects in *Drosophila*.

### Limits of standing variation in natural populations

We observe hundreds of segregating tandem duplicates in *D. yakuba* and *D. simulans*, spanning 2.6% of assayable the genome (i.e X and 4 major autosomal arms) in *D. yakuba* and 1.8% of the assayable genome in *D. simulans*. If evolutionary trajectories depend on tandem duplications to effect beneficial phenotypic changes, then these trajectories may be constrained if the population does not contain the desired variants as standing variation. We estimate the number of variants present in the entire population based on the observed sample variation in order to determine the extent to which selection will be limited by mutation. We estimate that the population contains at most 6800 segregating tandem duplications in *D. yakuba* and 4,500 in *D. simulans* (Tables E-F in S1 File), corresponding to 13.4% of major chromosome arms in *D. yakuba* and 9.7% of major chromosome arms in *D. simulans*. Estimates using rarefaction estimators free from assumptions of neutrality [57] are comparable (Table E in S1 File). Thus, the standing variation for tandem duplications will be insufficient to offer tandem duplications for every potential mutation for the majority of the genome (≈ 85%). If a tandem duplication is required for adaptation, evolutionary trajectories must then by definition rely on new mutations. Tajima’s *D* is negative in both *D. yakuba* and *D. simulans* suggesting recent population expansion and greater census size than effective population size. Even under expectations of large population sizes of 10^8^ after population expansion, we estimate that there are at most 8550 duplications segregating in the population at large for *D. yakuba* and 5700 for *D. simulans* (Table E in S1 File), still far from expectations required to span the entire genome (Table F in S1 File).

We calculate population level mutation rates *θ*_*π*_ (4*N*_*e*_ *μ*) of 0.00277 per gene per generation for whole gene duplications, 0.00082 for recruited non-coding sequence and 0.00088 for chimeras in *D. yakuba*. Population level mutation rates in *D. simulans* are slightly higher for most types of mutations with 0.00291 per gene per generation for whole gene duplications, 0.00117 for recruited non-coding sequence but a lower population level mutation rate of 0.00041 for chimeras. In comparison, we calculate *θ*_*π*_ for putatively neutral intronic SNPs of 0.0138 for *D. yakuba* and 0.0280 for *D. simulans*. We use these estimates of *θ* to calculate the likelihood of adaptation from alleles among the standing variation rather than new mutation for a population (*P*_*sgv*_) [18] assuming variants with a large selection coefficient of 1% under an additive genetic model. With such low levels of *θ*_*π*_ the likelihood of adaptation from a tandem duplication among the standing variation is 2.2% in *D. yakuba* and 2.6% in *D. simulans* (Table 1), a strikingly low likelihood that standing variation offers a sufficient substrate for adaptation. Even with a massive selective coefficient of *s* = 0.20 [24], the likelihood of adaptation from standing variation rather than new mutation is 3.1% for duplicates in *D. yakuba* and 3.4% in *D. simulans*. Chimeras are even more extreme with less than a 1% chance of fixation from standing variation (Table 1). In comparison, intronic SNPs have a likelihood of adaptation from standing variation of 12.1% in *D. yakuba* and 24.6% in *D. simulans* given s = 0.01, and 15.7% in *D. yakuba* and 30.1% in *D. simulans*, given extreme selection coefficients of s = 0.20 (Table 1). Thus, the limits of standing variation are expected to be far more severe for complex gene structures than for SNPs and will not offer sufficient standing variation to provide for adaptation via tandem duplication for any randomly selected gene.

**Table 1:** Mutation limited evolution in *D. yakuba* and *D. simulans*.

Species		Intron SNPs	Whole Gene	Recruit*	Chimera**
*D. yakuba*	*μ*	5.8 × 10^−9^	1.17 × 10^−9^	3.46 × 10^−10^	3.70 × 10^−10^
	*θ*_*π*_	0.0138	0.00277	0.00082	0.00088
	*P*_*sgv*_, s = 0.01	12.1%	2.23%	0.67%	0.71%
	*P*_*sgv*_, s = 0.20	15.7%	3.05%	0.91%	0.97%
	*T*_*e*_, s = 0.01	7270	36,000	122,000	114,000
	*T*_*e*_, s = 0.20	364	1,800	6,087	5,704
Species		Intron SNPs	Whole Gene	Recruit	Chimera
*D. simulans*	*μ*	5.8 × 10^−9^	6.03 × 10^−10^	2.42 × 10^−10^	8.52 × 10^−11^
	*θ*_*π*_	0.0280	0.00291	0.00117	0.00041
	*P*_*sgv*_, s = 0.01	24.6%	2.56%	1.04%	0.37%
	*P*_*sgv*_, s = 0.20	30.1%	3.41%	1.38%	0.49%
	*T*_*e*_, s = 0.01	3580	34,400	85,700	243,000
	*T*_*e*_, s = 0.20	179	1,720	4,290	12,100

* Tandem duplications that recruit non-coding sequence to form new genes.

** Chimeric genes formed through tandem duplication.

*P*_*sgv*_ from Hermisson and Pennings (2005) estimates the likelihood of adaptation from standing genetic variation under an additive model assuming neutral variation.

*T*_*e*_ (Gillespie 1991 and Maynard Smith 1971) estimates the average time until establishment of a selective sweep from a new mutation in generations given that a site is under strong selection with beneficial mutation rate equal to *θ*_*π*_. Estimates provide a lower bound on *T*_*e*_.

*Drosophila* have large effective population sizes and should offer large absolute numbers of tandem duplications in comparison to many other multicellular eukaryotes. However, the dynamics of standing variation may be drastically different for organisms with varying effective population sizes. We estimate time to loss given loss (sometimes called the sojourn time) [58], population mutation rates, and maximum nearly-neutral selection coefficients [59] for organisms with different population sizes in order to determine the extent to which dynamics observed in *Drosophila* might be applicable to other organisms. For organisms with very low *N*_*e*_, we expect to observe fewer mutations due to lower population level mutation rates, but a greater tolerance for extreme variation as more variants are subject to nearly neutral dynamics though sojourn times are largely unaffected (Table I in S1 File). Under such a scheme, evolution will still be severely limited by mutation with only small numbers of mutations appearing in the population. Given small *N*_*e*_, however, an extreme variant needed for adaptation to extreme environmental change is more likely to be tolerated among the standing variation. Thus, there may be higher variance in adaptive outcomes for organisms with small *N*_*e*_ due to the limits of mutation and in extremely rare cases organisms with small *N*_*e*_ might be able to adapt to sudden and drastic shifts in selective pressures.

### Long waiting times for new mutations

**Fig 5:**
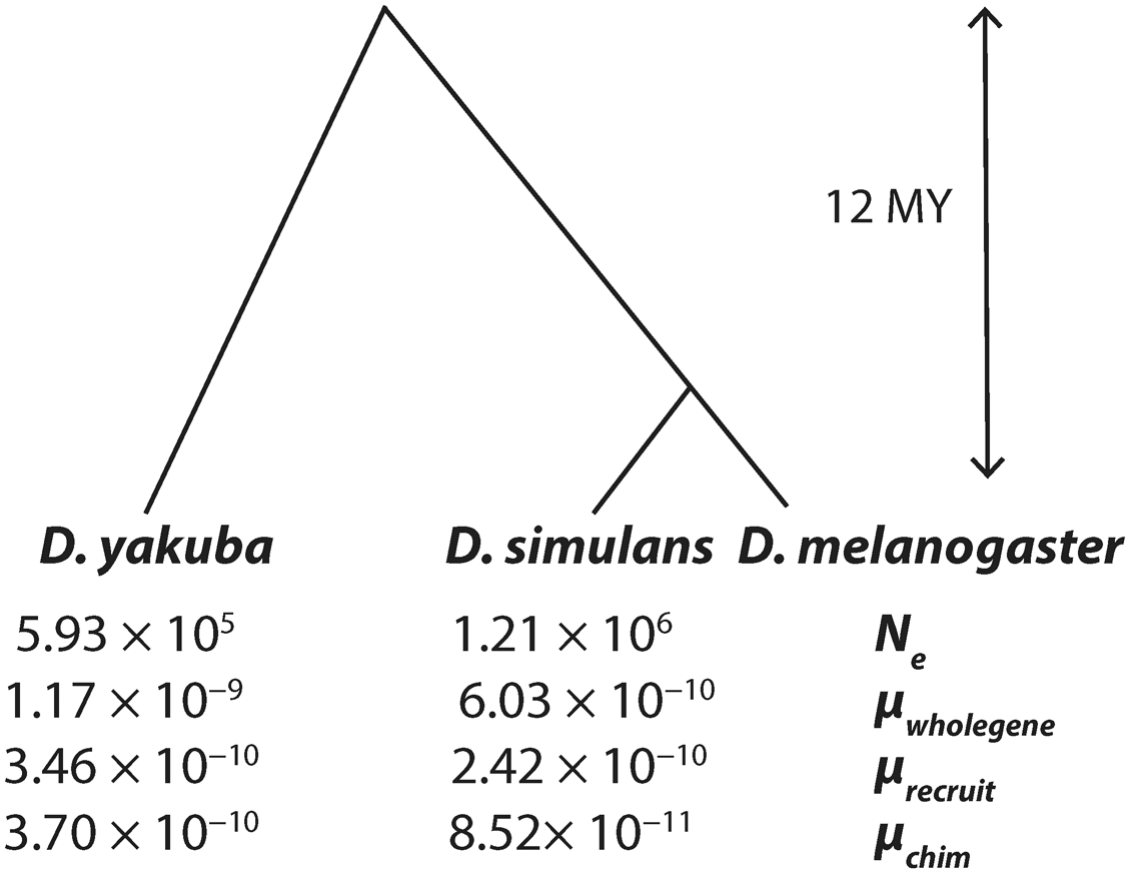
Genomewide population mutation rates for all duplications (*θ*), population sizes (*N*_*e*_), and per gene mutation rates (*μ*) for gene structures produced by whole gene duplication, recruitment of non-coding sequence, and chimeric genes by species. Low mutation rates and mutation limited evolution leads to low levels of parallel recruitment of tandem duplications.

We calculate the per generation effective mutation rate *μ* per gene for whole gene duplications, considering duplicates that capture 90% or more of gene sequences, in agreement with previous methods [10]. We estimate a whole gene duplication rate of 1.17 × 10^−9^ per gene per generation for *D. yakuba* and 6.03 × 10^−10^ per gene per generation for *D. simulans* (Fig 5, Table 1). These estimates are in general agreement with surveys of duplicates in the *D. melanogaster* reference genome of 3.68 × 10^−10^ per gene per generation [8, 10]. The rate of recruited non-coding sequence is 3.46 × 10^−10^ in *D. yakuba* and 2.42 × 10^−10^ in *D. simulans* and the rate of chimeric gene formation is equally low with 3.7 × 10^−10^ in *D. yakuba* and 8.52 × 10^−11^ in *D. simulans* (Fig 5, Table 1). We observe more tandem duplications in *D. yakuba* in spite of its lower *N*_*e*_, yielding a duplication rate per gene in *D. yakuba* two-fold higher than that of *D. simulans*. New mutations are often unable to spread through populations as low frequency variants can be lost through stochastic drift especially if recessive [60]. Therefore, the time to a sweep on a new mutation is expected to be substantially longer than the time until new mutations appear in populations [19, 20].

Given these estimates of *θ*_*π*_ for each class of mutations, we estimate *T*_*e*_, the time to establishment of a deterministic sweep from new mutations in a population such that variants overcome the forces of drift [19, 20]. We assume that beneficial mutations appear at strongly selected sites at a rate equivalent to the genome-wide effective mutation rate. In reality not all mutations are beneficial and the true rate of adaptive substitution is likely to be less common than those discussed here. These estimates therefore represent a lower bound on the time to adaptation through new mutation. With a modest selection coefficient of s = 0.01 similar to that previously observed for duplicates and chimeras [12], in *D. yakuba* *T*_*e*_ would be 7270 generations (600 years at 12 generations per year) for SNPs, 36,000 generations (3000 years) for whole gene duplications, and over 100,000 generations (≥ 9500 years) for chimeric genes (Table 1). For *D. simulans*, these numbers point to a greater disparity between SNPs and duplicates with *T*_*e*_ of 3580 generations (300 years) for SNPs, 34,400 generations (2800 years) for whole gene duplications, and 243,000 generations (20,000 years) for chimeric genes (Table 1). These estimates of effective mutation rates for whole gene duplications and complex gene structures point to long waiting times for new mutations and a disparity in the response of duplicates and SNPs in the face of strong selective pressures. Although the differences in effective mutation rates appear to be modest, they can result in additional thousands of years in the waiting time for selective sweeps to establish with new mutations, resulting in limited ability to adapt to shifting selective pressures.

Under more extreme selection coefficients, given the assumption that the beneficial mutation rate matches the mutation rate per site, waiting times may be shorter, allowing for adaptation at SNPs in hundreds of generations (decades) and thousands of generations (centuries) for gene duplications (Table 1). Under expanded census sizes 100X larger than *N*_*e*_ with extreme selection coefficients, waiting times for new mutations may potentially approach the range of full availability of mutations (Table H in S1 File). However, such extreme dynamics are unlikely to reflect the range of selection coefficients or the rate of adaptation genomewide [61, 62] and are well outside selection coefficients previously estimated for duplicate and chimeric genes of ≈ 1% [12]. Thus, we would not expect such estimates based on selection coefficients of 20% to be broadly applicable.

### Parallel evolution for tandem duplications

We find 56 genes are partially or wholly duplicated both in *D. yakuba* and in *D. simulans*, 11% of duplicated genes in *D. simulans* ($\frac{56}{478}$) and less than the number of genes duplicated multiple times in *D. simulans* alone, suggesting that there is little concurrence in the standing variation of the two species. That 56 genes would be shared across the two species is greater than expected given the limits of available standing variation of 478 duplicated genes in *D. simulans* and 875 in *D. yakuba* based on uniform chance (*P* = 2.812 × 10^−8^, binomial test) pointing to mutational or selective pressures on similar genes (SI Text). Fewer annotated gene models are available for *D. simulans* *w501* reference [63] leading to smaller absolute numbers of genes even though proportions are similar. Furthermore, a comparison to duplicate genes in *D. melanogaster*[64] shows only 5 genes that exist among the segregating variation of tandem duplications in all three species. The mutations described here have been polarized with respect to ancestry, and are segregating meaning that they are expected to have formed very recently. As such, shared variants are the product of independent mutation in the two species, not shared ancestry. We find that 13.4% of the genome is present but unsampled in *D. yakuba* and 9.7% in *D. simulans*, indicating that the likelihood of shared, unsampled variation is low. Such unsampled alleles will be at low frequency and are unlikely to be able to establish selective sweeps. Hence, the portion of variation available for selective sweeps that is shared across species will be low, resulting in a rarity of evolution through parallel recruitment of tandem duplicates.

Some genes within the genome are captured by as many as 6 independent tandem duplications in *D. yakuba* and 32 independent tandem duplications in *D. simulans*. There are 10 genes in *D. yakuba* and 12 genes in *D. simulans* that are captured by more than three independent tandem duplications, and these have been excluded from mutation estimates (Table G in S1 File). Some of these variants are chorion proteins known to experience somatic duplications in follicle cells [65], and certain of these hotspots may therefore represent cases of somatic mutation rather than inherited variation. These “hotspots” within the genome may have duplication rates high enough that evolution will not be subject to the same limitations with respect to standing variation and sweeps on new mutations.

### Rapid Evolution

**Fig 6:**
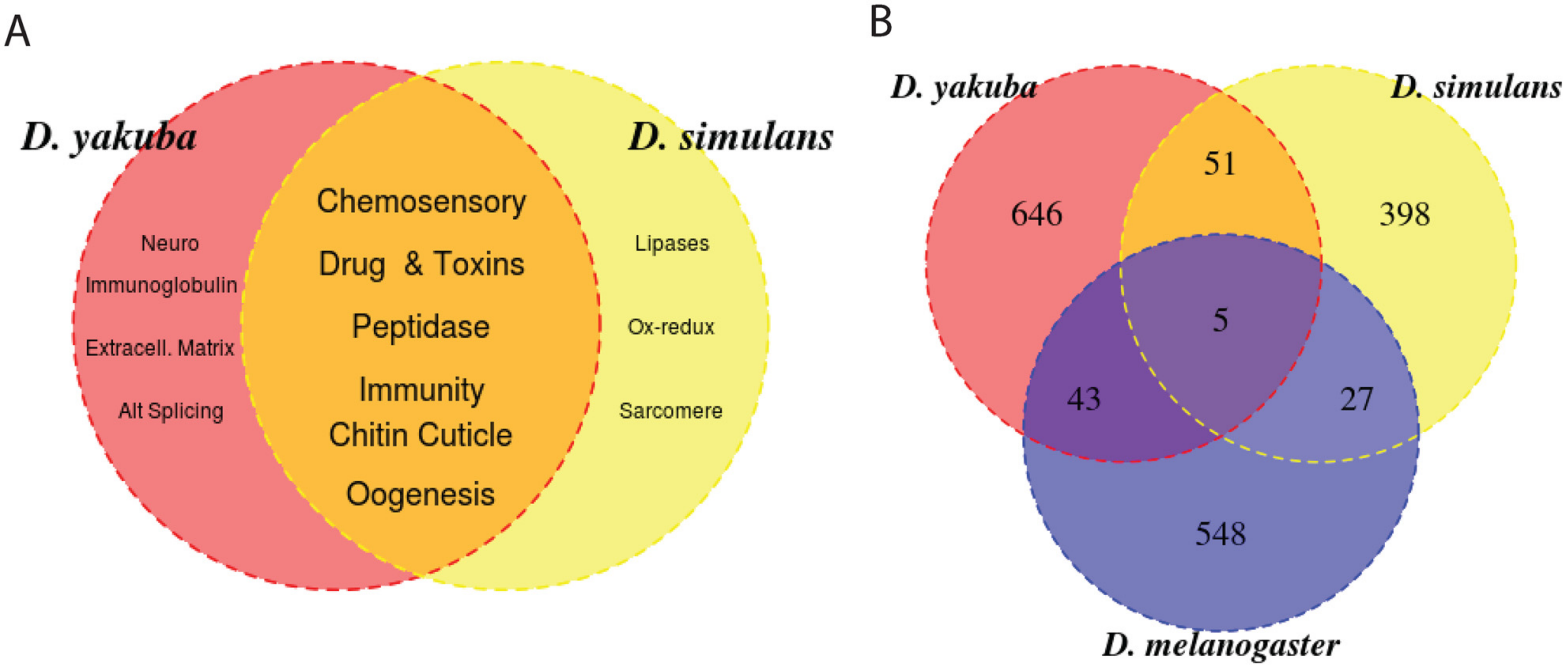
A) Gene ontology classes overrepresented by species among singly duplicated genes or among multiply duplicated genes. B) Number of genes duplicated by species. Most variants are species specific, with small numbers of parallel duplication of orthologs across species.

Biases in the rates at which tandem duplications form in different genomic regions or a greater propensity for selection to favor duplications in specific functional classes can result in a bias in gene ontology categories among duplicated genes. We previously used DAVID gene ontology analysis software to identify overrepresented functions among duplicate genes in *D. yakuba* and *D. simulans* [4]. Here, we compare the agreement in gene ontology categories in *D. yakuba* and *D. simulans* with expectations based on random sampling to determine whether such convergence at the level of functional categories is significant. Notably, among randomly selected subsets of genes for *D. yakuba* and *D. simulans* there is no agreement in functional categories at an EASE cutoff of 1.0 for any biochemical function or domain, and no functional category was significant at an EASE cutoff of 1.5, a stark contrast with what is observed for duplications (see S2 File). Among genes captured by tandem duplications, immune response and toxin metabolism, chitin cuticle formation and chemosensation are overrepresented in both species (Fig 6A, Table D in S1 File). Such overrepresentation is not identified in randomly selected subsets of genes for *D. yakuba* and *D. simulans*, suggesting a greater level of convergence than is expected based on chance alone (Table D in S1 File). To determine whether selection is favoring these functional classes, we identified duplications outside centromeric regions that lie in windows at or below the 5% tail of *θ*_*π*_, consistent with selection reducing diversity. Among these genes in regions with reduced diversity we identify genes in both *D. yakuba* and *D. simulans* with functions in chorion or oogenesis, mating behavior, immune response and defense against bacteria, olfactory response, chitin metabolism, xenobiotics and toxin metabolism, and sperm development (Supplementary Information). The presence of genes with these functional categories is consistent with a portion of the overrepresentation in gene ontologies across all duplicates being driven at least in part by selection.

## Discussion

We have described the prevalence of tandem duplications in natural populations of *D. yakuba* and *D. simulans*, their frequencies in the population, and the genes that they affect. We find that tandem duplications show a bias towards gene ontologies associated with rapid evolutionary processes and that they commonly affect the X chromosome in *D. simulans* in comparison to the autosomes. In spite of their strong role in adaptation, we find low rates of parallel recruitment of tandem duplications across species due to low formation rates and mutation limited evolution.

### Widespread positive selection on the X chromosome in *D. simulans*

We observe an excess of high frequency tandem duplications on the *D. simulans* X chromosomes in comparison to neutral intronic SNPs as well as signs of reduced diversity surrounding tandem duplications on the *D. simulans* X, consistent with widespread selection. Background selection [66] and hitchhiking [67] are not expected to act differently on duplications in comparison to SNPs and cannot explain the patterns observed. Yet, we observe significant differences between the SFS of duplicates and putatively neutral SNPs, pointing to a role for adaptation through tandem duplication. We also observe reduced nucleotide diversity surrounding tandem duplications on the *D. simulans* X, consistent with selection favoring duplicates. Hence, the overabundance of high-frequency tandem duplications on the X is likely to be driven by selection and these represent strong candidate loci for ongoing selective sweeps. Based on the newly assembled *D. simulans* reference, X vs. autosome divergence indicates faster evolution on the X chromosome at non-synonymous sites, long introns, and UTRs [63]. This pattern is distinct from observations at synonymous sites as well as general patterns of differential evolution on the autosomes [63], further evidence of more frequent selective sweeps on the X chromosome. Roughly 25% of tandem duplications in each species are flanked by repetitive sequence and there is an overabundance of tandem duplicates associated with repetitive sequence on the *D. simulans* X [4]. It is possible that such repeats contribute to the formation of these mutations, thereby influencing the evolution of the *D. simulans* X.

The X chromosome is thought to evolve rapidly due to sexual conflict, intragenomic conflict, and sexual selection [68] and thus multiple selective forces may facilitate the spread of duplicates on the X. The X chromosome in *D. simulans* houses an excess of duplicates in comparison to all autosomes, as well as a strong association with repetitive sequence and tandem duplications on the X [4]. Therefore, the X chromosome appears to be subject to particularly rapid evolution of duplicate content in *D. simulans*. Previous work has identified signals of adaptation through duplication on the *D. melanogaster* X chromosome [69, 70], suggesting parallel evolution through duplication in these species. However, we do not observe similar patterns in *D. yakuba*, suggesting that the X may either be evolving under different selective pressures in the two different clades or that selective pressures on the *D. yakuba* X chromosome are of lesser magnitude. Stronger sexual selection, greater selection for X-chromosome related traits, sympatric associations with competitor species with reinforcement for mating aversion, or a greater instance of driving X chromosomes might potentially drive these species differences in X-chromosome evolution, and elucidating the nature of selection on these sex chromosomes may help explain the adaptive (or selfish) role of tandem duplication on the X.

### Mutation limited evolution

While both *D. simulans* and *D. yakuba* house a rich diversity of duplicated sequences, only a few percent of the genome will be covered by tandem duplications. With lower mutation rates for duplications [8, 10, 71], there may be long waiting times to achieve any single new mutation, and the landscape of standing variation will shape evolutionary outcomes. As such, any evolutionary path that is dependent upon tandem duplications of any specific genomic sequence will be severely limited by the small likelihood that the necessary mutation is among the standing variation. *Drosophila* represent organisms with large effective population sizes (Fig 5) [22, 72] and are expected to host large numbers of duplications as standing variation in comparison to other multicellular eukaryotes. We have shown that the number of tandem duplications segregating in the population is substantially smaller than the number of mutations needed to guarantee a duplicate of any desired genomic region. However, when population level mutation rates are small, standing variation is unlikely to offer a sufficient substrate for selective sweeps and systems will be stuck waiting for new mutations that are slow to materialize [18]. We observe population level mutation rates *θ* per gene for tandem duplications on the order of 0.00277 in *D. yakuba* and 0.00291 in *D. simulans* (Table 1, Fig 5) resulting in low probabilities that standing variation offers the major source of adaptation and long waiting times to sweeps on new mutations on the order of hundreds to thousands of years. While retrogenes might provide additional sources of duplicated sequences, their rates of formation are exceptionally limited [8, 10] and they are therefore not expected to contribute more substantially than tandem duplications to genomic variation and will not suffice to overcome these limitations of low mutation rates. Thus, we conclude that outside of a small number of mutational hotspots evolution through duplication is mutation limited even in *Drosophila* which have large *N*_*e*_, and that these limits are expected to be even more severe for many other multicellular eukaryotes, especially vertebrates. In some rare cases, organisms with small *N*_*e*_ may harbor more extreme variation under nearly neutral dynamics, but the absolute number of such mutations will be very limited. Thus, cases of adaptation from standing variation of extreme variants in organisms with small *N*_*e*_ are possible, but rare.

The majority of tandem duplications identified in *D. yakuba* and *D. simulans* appear to be at extremely low frequency, with an excess of singleton variants in comparison to neutral intronic SNPs, suggesting that large numbers of tandem duplications are detrimental, consistent with previous work in other species [5]. It has previously been argued that the accumulation of duplications is the product of small *N*_*e*_ and inability of selection to purge nearly neutral alleles from the population [17, 59]. However, we show that duplicates are less likely to be neutral in comparison to putatively neutral intronic SNPs suggesting that they often have phenotypic effects larger than the limit near-neutrality. We have shown that both positive and negative selection will affect the fixation or loss of duplications and that simplified nearly neutral theories are unlikely to explain the patterns observed across species. Rather, selection is expected to play an appreciable role in the evolution of tandem duplications and their contribution to genome content. Previous work has shown different patterns for young and old duplicate genes, with many young duplicates and chimeras forming in tandem but more duplicates and chimeras preserved over long periods that are where paralogs are dispersed from one another [8, 10, 73] even though variants found in tandem show signals of selection. Two factors are likely to explain this disparity. First, genome shuffling and syntenic breaks are common over time [74, 75]. Second, it is likely that forces leading to adaptation are likely to be distinct from forces that lead to gene preservation as preservation requires that genes remain essential over long periods of time, especially if selective pressures on non-essential genes are transient [10, 12].

### Likelihood of parallel recruitment of tandem duplications across species

Convergent evolution is often interpreted of a signal of adaptation in experimental evolution and in natural populations [29, 30]. Here, we show that for tandem duplications, parallel recruitment of genes for duplication and diversification independent from shared ancestry will be very rare in spite of convergence in functional categories represented. Thus, the reliance on genetic convergence to establish natural selection in natural populations will underreport selected alleles and result in significant underestimation of the number and types of alleles that are selected. Though convergence is common in experimental evolution of both prokaryotic systems and multicellular eukaryotes with shared ancestry [30], these results suggest that these systems are unlikely to reflect the frequency of convergent evolution in natural populations of independently evolving species of multicellular eukaryotes that have little shared standing variation. We observe an excess of variants with gene ontologies consistent with similar rapid evolutionary processes both in *D. yakuba* and in *D. simulans* (Fig 6A). However, few genes (∼ 11%) are duplicated in both species and only a handful have been identified in *D. simulans*, *D. yakuba*, and *D. melanogaster* (Fig 6B). Moreover, none of the high frequency variants in the in *D. yakuba* and *D. simulans* capture orthologous sequences. Hence, in spite of parallel selective pressures on rapidly evolving phenotypes, there is little parallel recruitment of the same genetic solutions with respect to duplication. Given the limited genomic span of standing variation in the population (Table F in S1 File), and low rates of new mutation (Fig 5, Table 1), as well as the low frequency of a large fraction of variants, parallel fixation of tandem duplications in the same genes will be extremely rare even among genera with large effective population sizes facing similar selective pressures.

Convergence depends on the waiting time of new mutations to enter populations and establish selective sweeps. We show that the average waiting time for a new mutation given a selection coefficient of *s* = 0.01 is hundreds of years for SNPs. Here, we find that tandem duplications display signals of reduced heterozygosity in the surrounding regions as well as an association with gene ontologies indicative of rapidly evolving phenotypes, and an overrepresentation of shared tandem duplicates across species for specific genes given the limits of standing variation, consistent with widespread adaptation through tandem duplication. However, the average waiting time for a deterministic sweep to establish in a population will be thousands of years for tandem duplications and tens of thousands of years for chimeric genes given a modest selection coefficient of *s* = 0.01. Such strongly selected sites are expected to be rare throughout the genome and beneficial mutations are likely to appear less often than the actual mutation rate [61, 62]. Thus, these waiting times given strong selection provide a lower bound to the waiting time for a selected sweep. We therefore expect that mutation will severely limit evolution through whole gene duplication and chimera formation. To the extent that adaptation depends on tandem duplications, the ability of organisms to adapt to changing environments will be hindered by a lack of variation. Thus, even when a given tandem duplication is needed for adaptation, we expect that the limits of mutation will lead to low levels of convergence and scarcity of shared genetic solutions.

### Duplicate genes and rapidly evolving phenotypes

Both *D. simulans* and *D. yakuba* have an overabundance of genes with ontology classifications involved in immune function, chemosensory processing or response, and drug and toxin metabolism that is significantly greater than expectations based on random chance (Table D in S1 File). These phenotypes are strongly associated with rapid evolution due to host-parasite interactions, predator-prey coevolution, and sexual conflict [76–79]. Previous work has observed similar bias toward rapid amino acid substitutions in olfactory genes, and chitin cuticle genes in *D. melanogaster* and *D. simulans* [80], selection for gene family evolution in and selection for toxin resistance is common in *D. melanogaster* [78, 81] suggesting that associated phenotypes may be under widespread selection in multiple species.

Host pathogen systems as well as arms races in pesticide and toxin resistance operate under Red Queen dynamics in which conflicts between organisms result in repeated selective sweeps [82]. Organisms that lack the genetic means to adapt to rapidly changing systems will be at a distinct disadvantage in the face of selective events. Additionally, the overrepresentation of tandem duplications in cytochromes and drug or toxin metabolism genes confirms rapid evolution in copy number seen in comparison of reference genomes [75] as well as recent studies of insecticide resistance and viral resistance in natural populations [3, 81, 83]. Large amounts of divergence driven by selection among non-synonymous sites and UTRs in *D. simulans* [84] and high rates of adaptive substitutions [80, 85] point to widespread selective pressures acting in *D. simulans*, and it is likely that these same pressures influence the current diversity and frequency of copy number variants. If rapidly evolving systems rely heavily on complex mutations or if selection coefficients are modest, profiles of standing variation will place strong limits on outcomes in response to selection.

Shifting selective pressures such as those found in rapidly evolving systems or gross ecological change require a pool of genetic variation to facilitate adaptation. We observe standing variation and mutational profiles that will limit evolutionary trajectories and would expect these limits to be even more severe for rapidly evolving phenotypes. Repeated sweeps are expected to purge genetic and phenotypic diversity, and recovering such diversity after sweeps can take thousands of generations [86]. Thus, during rapid evolution, selection will potentially purge diversity that is needed for subsequent steps in the adaptive walk. Hence, although tandem duplications are key players in rapid evolution, their limited rates of formation combined with low frequencies due to commonly detrimental impacts will hinder evolutionary outcomes or force alternative adaptive trajectories precisely when variation is urgently needed. Hence, we do not observe convergence across individual loci in spite of substantial convergence across functional categories.

## Materials and Methods

### Tandem duplications

Tandem duplications were identified using paired-end Illumina sequencing of genomic DNA for 20 strains of *D. yakuba* and 20 strains of *D. simulans* as well as the reference genome of each species as described in Rogers et al. (2014). The dataset describes derived, segregating tandem duplications that span 25 kb or less. These sequences exclude ancestral duplications as well as putative duplications in the resequenced reference genomes. The resulting list of variants describes segregating variation for newly formed tandem duplicates across the full genome in these two species of non-model *Drosophila*. All data files are available via http://molpopgen.org/Data and http://www.github.com/ThorntonLab/DrosophilaPopGenData-Rogers2014. Aligned bam files were deposited in the National Institutes of Health Short Read Archive under accession numbers SRP040290 and SRP029453. Sequenced stocks were deposited in the University of California, San Diego (UCSD) stock center with stock numbers 14021-0261.38- 14021-0261.51 and 14021-0251.293–14021-0251.311.

### Identifying duplicated coding sequence

Tandem duplications were previously identified using a combination of paired-end read mapping and coverage changes in 20 isofemale lines of *D. yakuba* and 20 isofemale lines in *D. simulans* generated via 9–12 generations of sibling mating from wild-caught flies. We sequenced 10 isofemale lines of *D. yakuba* from Nairobi, Kenya, and 10 isofemale lines from Nguti, Cameroon as well as 10 isofemale lines of *D. simulans* from Nairobi, Kenya and 10 isofemale lines from Madagascar. Duplications were identified through divergently oriented reads and coverage changes in comparison to reference genomes. We identify 1415 tandem duplications in *D. yakuba* and 975 tandem duplications segregating in *D. simulans* that span 845 different gene sequences in *D. yakuba* and 478 different gene sequences in *D. simulans* [4]. Gene duplications were defined as any divergent read calls whose maximum span across all lines overlaps with the annotated CDS coordinates. *D. yakuba* CDS annotations were based on flybase release *D. yakuba* r.1.3. Gene annotations for the recent reassembly of the *D. simulans* reference were produced by aligning all *D. melanogaster* CDS sequences to the *D. simulans* reference in a tblastx (http://genomics.princeton.edu/AndolfattoLab/w501_genome.html). Percent coverage of the CDS was defined based on the portion of the corresponding genomic sequence from start to stop that was covered by the maximum span of divergent read calls across all strains. Using the representation of gene sequences in *D. yakuba* of $\frac{845}{16082}$ we use a binomial test to calculate the likelihood of 56 shared variants among the $\frac{478}{10786}$ genes duplicated in *D. simulans*.

### Estimated number of segregating tandem duplications

We compared the estimated total number of duplications expected in a population to estimates of diversity based on our sample of 20 strains, correcting *S* for a 3.9% false positive rate (Table E in S1 File). Under a standard coalescent model [50, 87, 88]:

  $$E[S_{population}]=\frac{S_{sample}}{a_{sample}}*a_{population}$$
Where a in a sample of size n (in this case n = 20):
  $$a_{sample}=\sum_{i=1}^{n-1}\;\frac{1}{i}$$
  $$a_{population}=\sum_{i=1}^{2N_{e}}\;\frac{1}{i}$$
When 2*N*_*e*_ is large:
  $$\theta \sum_{i=1}^{2N_{e}}\;\frac{1}{i}\approx \theta (ln(2N_{e})+0.57722)$$
Hence:
  $$E[S_{population}]=\frac{S_{sample}}{a_{20}}*(ln(2N_{e})+0.57722)$$
We can use similar methods to estimate the variance in the number of segregating sites in the population.
  $$Var[S_{population}]=\theta \sum_{i=1}^{2N_{e}}\;\frac{1}{i}+\theta^{2}\sum_{i=0}^{2N_{e}}\;\frac{1}{i^{2}}$$
When 2*N*_*e*_ is large:
  $$\sum_{i=1}^{2N_{e}}\;\frac{1}{i^{2}}\approx \frac{\pi^{2}}{6}.$$
  $$Var[S_{population}]=\theta (ln(2N_{e})+0.57722)+\theta^{2}\frac{\pi^{2}}{6}$$

Alternatively, we can use rarefaction estimators, which are free of population genetic assumptions to estimate the total number of duplications. Using the Chao estimator [57] for $S_{total}=S_{obs}+\frac{{S_{1}}^{2}}{2S_{2}}$, where *S*_1_ and *S*_2_ are the number of variants at a frequency of 1 and 2 resepectively. The estimated variance is then $S_{2}[{\left(\frac{S_{1}}{4S_{2}}\right)}^{4}+{\left(\frac{S_{1}}{S_{2}}\right)}^{3}+{\left(\frac{S_{1}}{2S_{2}}\right)}^{2}]$.

### Gene Ontology

Overrepresented functional categories were identified using DAVID gene ontology software with an EASE threshold of 1.0. as previously described [4]. We observe several functional categories indicative of rapid evolution that are shared between the two species (Table D in S1 File). In order to determine whether such agreement at the level of functional category is greater than expected by chance, we selected a random subset of 845 genes for *D. yakuba* and 478 genes from *D. simulans*, and performed ontology analysis for a comparison.

### Proportion of the genome represented by segregating duplicates

To determine the number of duplications necessary to span the full range of the genome, we simulated chromosomes with a length determined by the number of base pairs with non-zero coverage in our reference strain. We then simulated random draws from the distribution of duplication lengths for each chromosome, placing duplication start sites at random and recorded the number of duplications necessary to cover 10%, 25%, 50%, and 90% of sequence length for each chromosome in each trial. Simulations were repeated for 1000 trials for each chromosome.

These simulations do not account for mutational biases that might result in clustering of duplications in particular regions while other regions remain static, nor do they require that new duplications reach an appreciable frequency so that they are immune to stochastic loss through genetic drift. They do not require that duplications capture sufficient sequence to have functional impacts or require that breakpoints not disrupt known functional elements. Furthermore, simulating individual chromosomes separately decreases the likelihood of resampling particular sites thereby lowering the estimated number of duplications needed to cover the entire genome. Hence, these estimates put a highly conservative lower bound on the minimum number of mutations necessary to capture the full genomic sequence.

To estimate the expected proportion of the genome spanned by all duplicates in the population, we resampled 6700 duplicates from the observed size distribution of *D. yakuba* with replacement and 4000 duplicates from the observed size distribution of *D. simulans*, placing duplications at random positions across the chromosome. We performed 100 replicates of sampling and report the mean across all replicates for each species. In *D. simulans* we observe one case with 19 independent whole gene duplications of a single ORF [4], suggesting up to 1000-fold variation in mutation rates over the genome average. Estimates of population level variation and genome wide effective mutation rates ignore mutation rate variation where some regions may be highly prone to duplications whereas others remain static, which would reduce the likelihood of unobserved tandem duplications outside of mutational hotspots. Hence, these estimates represent a lower bound on the number of duplications necessary to span the entire genome.

### Effective mutation rates and waiting times for duplicates

We estimate average heterozygosity (*θ*_*π*_) and effective mutation rates (*μ*) per gene for *D. yakuba* and *D. simulans* for gene duplications that capture at least 90% of gene sequence (in agreement with previous estimates [10]), for genes that recruit non-coding sequence, and for chimeric genes. Heterozygosity estimates used to calculate effective mutation rates were corrected for ascertainment bias (see SI Text) and excluded genes that were captured by 4 or more independent mutations, a signal of hotspots and mutation rate heterogeneity. Heterozygosity per gene is estimated given 16,082 gene sequences in *D. yakuba* and 10,786 coding sequences in *D. simulans* (Table 1). Like all estimates of mutation rates, these will exclude lethal varinats or variants that produce sterility or early-life pathogenic effects. However, they should accurately reflect the amount of variation that can be observed among standing variation, including even moderately deleterious mutations that are destined for eventual loss. Given estimates of *θ*_*π*_, we estimate the probability of adaptation from standing variation under an additive genetic model for neutral variants, *P*_*sgv*_ = 1 − *e* ^−*θ*_π_*ln(1+2*N*_*e*_*s*)^ [18] and the time to establishment (*T*_*e*_) of a deterministic sweep from new mutations, such that new mutants escape the stochastic forces of drift, $T_{e}=\frac{1}{\theta_{\pi }s}$ [19, 20]. These estimates are provided for two strong selection coefficients of *s* = 0.01 similar to what is observed in *Drosophila* for chimeric and duplicate genes [12] and *s* = 0.20 modeling abnormally strong selection on a single locus consistent with Karasov et al. [24]. Estimates assume that a given site of interest is under strong selection and that the beneficial mutation rate is equal to the mutation rate per site per generation providing an upper limit on the ability of new mutations to facilitate adaptation. In reality, strongly selected sites will be rare throughout the genome [61, 62] and these waiting times given strong selection will not accurately reflect the expected number of selective sweeps throughout the genome. We additionally estimate the time to loss given loss for alleles in the population according to 2*ln*(*N*_*e*_) [58], the nearly neutral selection coefficient $s>\frac{1}{4N_{e}}$ [59] as well as population level mutation rates *θ* for alternative effective population sizes to determine the broader applicability of these results to other organisms.

### Intronic SNPs

In order to produce a neutral proxy for sequence change in each species, we identified SNPs for short introns 100 bp or less, focusing on sites 8–30 which are generally subject to little constraint [89–91]. Reads containing indels were re-aligned using GATK [92]. SNPs were identified across strains using samtools v1.18 mpileup [93] disabling probabilistic realignment (-B) and outputting genotype likelihoods in BCF format (-g). The resulting BCF used to create a VCF using bcftools, calling bases using Bayesian inference (-c) calling genotypes per sample (-g) with a scaled mutation rate of 1% (-t.01) under a haploid model (ploidy = 1). SNPs were required to have minimum Illumina coverage depth of 20 reads, maximum coverage of 250 reads, MQ ≥ 20, and GQ ≥ 30 and invar GQ ≥ 40. We excluded SNPs identified in the reference, which are indicative of either assembly errors or residual heterozygosity. We performed hierarchical cluster analysis in R using all SNPs by chromosome to evaluate population structure.

The ancestral state for each SNP was established through comparison with the nearest sequenced reference genome as an outgroup, *D. erecta* for *D. yakuba* sequences and *D. melanogaster* for *D. simulans* sequences. Orthologs between each species and its outgroup were identified using reciprocal best hit criteria in a BLASTn at an E-value cutoff of 10^−5^. Full gene sequences for each ortholog were then aligned using clustalw, keeping only genes which aligned with 85% or greater nucleotide identity. Divergence between the two species, *Div*_*x*, *y*_, was defined based on alignments of intronic sites from bases 8–30 between each species and the outgroup reference genome, excluding gapped sequences, for aligned orthologs with 85% or nucleotide identity. The ancestral state was defined based on the corresponding sequence in the outgroup genome (*D. melanogaster* for *D. simulans* and *D. erecta* for *D. yakuba*). We excluded sites where the outgroup reference was in disagreement with both the *D. yakuba* reference and *D. yakuba* SNPs, as well as triallelic SNPs, sites with reference sequence of ‘N’, or SNPs identified in the VCF for the reference, suggesting inaccuracies in reference assembly or residual heterozygosity in the reference. These resulted in a total of 7158 intronic SNPs in *D. yakuba* and 5504 intronic SNPs in *D. simulans*. The resulting unfolded SFS was then corrected for the probability of independent mutations in both reference genomes leading to incorrect inference of the ancestral state.

Parallel mutations occurring independently at nucleotide sites in different species can obscure evolutionary relationships and artificially skew SFS. We corrected SFS for SNPs prior to performing comparisons for tests of selection. Given net divergence *D*_*net*_ = *Div*_*x*, *y*_ − *π*_*x*_, the probability of identical independent mutations occurring in the outgroup reference genome is reflected by either the probability of an independent transition (ts) at the site of a transition mutation, or by 1/2 the probability of a transversion (tv) at the site of a transversion polymorphism. Thus,

$$k=\left[{\left(\frac{\kappa }{2+\kappa }\right)}^{2}+\frac{1}{2}{\left(\frac{2}{2+\kappa }\right)}^{2}\right]D_{net}$$ (1)

Empirically, in *Drosophila* $\kappa =\frac{ts}{tv}=2$. Thus, $k=\frac{3}{8}D_{net}$.

The unfolded SFS for intronic sites was corrected for the likelihood of independent mutations in the reference, k. The probability of independent mutations occurring in both genomes is equal to the probability of either two independent transitions or two independent transversions occurring in both genomes. We calculated *π*_*x*_ as the average heterozygosity per intronic site.

Given a likelihood of independent identical mutations of $k=\frac{3}{8}D_{net}$.

$$S_{i,obs}=E\left[S_{i}\right]-E\left[S_{i}\right]\left(k\right)+E\left[S_{n-i}\right]\left(k\right)$$ (2)

$$S_{n-i,obs}=E\left[S_{n-i}\right]-E\left[S_{n-i}\right]\left(k\right)+E\left[S-i\right]\left(k\right)$$ (3)

Substituting Eq 3 into Eq 2, we obtain

$$E\left[S_{i}\right]=\frac{S_{i,obs}\left(1-k\right)-S_{n-i,obs}\left(k\right)}{1-2k}$$ (4)

### Correcting Duplicates for Ascertainment Bias

Tandem duplications, unlike SNPs, cannot be identified using paired-end reads in individual strains except through comparison to the reference genome. Moreover, variants that are segregating at high frequency in populations are substantially more likely to be present in the reference, and therefore are substantially less likely to be identified in sample strains [5]. We corrected site frequency spectra according to the model developed previously [5].

$$x_{i}=\frac{y_{i}\frac{n}{n-i}}{\sum_{i=1}^{n-2}y_{i}\frac{n}{n-i}}$$ (5)

Here, *x*_*i*_ is the true proportion of alleles at frequency i in the population, and *y*_*i*_ is the observed proportion of alleles at frequency i in a sample of n strains (here 21). The correction for ascertainment bias lowers estimates of the proportion found a low frequencies and increases estimates of the proportion at high frequency. For estimates of population site frequency spectra, we removed all variants with divergently oriented reads in the reference strain, as these would not be identified in an accurately annotated reference.

### Residual heterozygosity

Some isofemale lines contained regions of residual heterozygosity in spite of over 10 generations of inbreeding in the lab. To detect regions of residual heterozygosity, we called SNPs as above under a diploid model. Segments with residual heterozygosity were detected using an HMM (“HMM”;http://cran.r-project.org/web/packages/HMM/).

Prior probabilities on states were set as:
  $$\pi =[\begin{array}{cc} 0.5. & 0.5 \end{array}]$$
Transition probabilities were set to:
  $$T=[\begin{array}{cc} 1-10^{-10} & 10^{-10}\\ 10^{-10} & 1-10^{-10} \end{array}]$$
and emission probabilities set to:
  $$E=[\begin{array}{cc} \theta & \epsilon \\ 1-\theta & 1-\epsilon \end{array}]$$

Where *ε* = 0.001 and *θ* = 0.01. The most likely path was calculated using the Viterbi algorithm, and heterozygous segments 10kb or larger were retained. Heterozygous blocks within 100kb of one another in a sample strain were clustered together as a single segment to define the span of residual heterozygosity within inbred lines.

### Differences in Site Frequency Spectra

If different classes of duplications have different selective impacts, we should observe clear differences in site frequency spectra, with more positively selected duplications showing fewer singleton alleles and more high frequency variants. Site frequency spectra are not normally distributed, nor can they be normalized through standard transformations, and thus require non-parametric tests. We used a two-sided Wilcoxon rank sum test to determine whether site frequency spectra were significantly different. For each comparison, we excluded tandem duplications that are present in the reference genomes as well as putative ancestral duplications, as these are likely to display biases with respect to size, propensity to capture coding sequences, and association with repetitive content. We compared site frequency spectra of the following groups within each species: duplications on the X and on the autosomes and all pairwise combinations of SNPs and duplicates on the X and autosomes. We also performed Kolmogorov-Smirnov test for comparison. In *D. simulans*, we used a *χ* ^2^ test to determine whether high frequency alleles are overrepresented among duplications on the X relative to intronic SNPs. Data for the *χ* ^2^ test was binned using a cutoff to compare the proportion of variants as at a sample frequency of $\geq \frac{16}{17}$.

Tandem duplicates that lie in regions with residually heterozygous segments extending 1kb upstream or downstream were excluded from the SFS, resulting in unequal sample sizes for different variants. Samples with fewer than 15 strains remaining were excluded from the SFS. The SFS for intronic SNPs and for duplicates was then scaled to a sample of size 17 in *D. simulans* and 15 in *D. yakuba* according to Nielsen et al. [48].

### Segregating Inversions

In order to check for population substructure, we aligned all SNPs in intronic sequences from 8–30 bp as a neutral proxy [89–91] and performed hierarchical clustering in R using hclust. These SNPs were intended solely to differentiate strains and were not polarized with respect to the ancestral state or otherwise filtered. We observe little evidence for population structure in *D. simulans* (Figure L in S1 File). However, we identify structure on chromosome 2 in *D. yakuba* (Figure M in S1 File), consistent with known polymorphic inversions prohibiting recombination on chromosome 2 [53, 54]. Strains do not strictly cluster with respect to geography but rather are reticulated amongst other groups. Moreover, among duplicates we do not observe an excess of moderate frequency alleles as one would expect under population substructure given our sampling scheme (Figure A in S1 File). Thus, these strains constitute a single admixed population.

Some strains retained residual heterozygosity even after 9 generations of inbreeding, with greater residual heterozygosity in *D. yakuba* than in *D. simulans*, consistent with inversions segregating in *D. yakuba*. These regions of residual heterozygosity can result in incorrect estimates of SFS by artificially increasing chances of observing variation. Site frequency spectra were calculated across all strais by correcting sample frequencies for ascertainment bias, excluding regions of residual heterozygosity and then projecting frequencies onto a sample size of 15 in *D. yakuba* and 17 in *D. simulans* according to [48]. As a neutral comparison we calculated SFS for intronic SNPs (as above) and projected the SFS down to a sample size of 15 in *D. yakuba* and 17 in *D. simulans* (Figure A in S1 File).

### Likelihood of shared variation through ancestry

The likelihood of shared variation through shared ancestry can be obtained through a coalescent approach. The probability that an allele does not coalesce in the time period from the present back to the speciation event that separated *D. yakuba* and *D. simulans* is ${\left(1-\frac{1}{2N_{e}}\right)}^{t}$. This can be approximated using $e^{\frac{-t}{2N_{e}}}$. We estimate *θ*_*pi*_ for putatively neutral 8–30 bp from short introns using libsequence [94], ignoring sites that are heterozygous and sites with missing data. For neutral intronic SNPs, *θ*_*pi*_ = 0.0138 in *D. yakuba* and *θ*_*pi*_ = 0.0280 in *D. simulans*. Using the mutation rate of 5.8 × 10^−9^ [95], we find *N*_*e*_ = (0.0138)/(4 × 5.8 × 10^−9^) = 5.93 × 10^5^ in *D. yakuba* and *N*_*e*_ = (0.0280)/(4 × 5.8 × 10^−9^) = 1.21 × 10^6^ in *D. simulans*. Using t = 12MY [28] and 12 generations per year, and *N*_*e*_ = 1.2 × 10^6^ from *D. simulans*, we obtain a probability of shared ancestry for an allele of 9 × 10^−27^, vanishingly small. We have polarized all mutations against the putative ancestral state using outgroup reference genomes, focusing solely on derived mutations [4]. Furthermore, the expectation of shared variation for any two alleles through shared ancestry for *D. yakuba* and *D. simulans* is expected to be low. Even large samples are not expected to harbor shared variation over such timescales [96]. Thus, we expect shared variants described here to result from independent mutations, not from long standing neutral polymorphism.

### Diversity surrounding SNPs and duplications

We estimate *θ*_*π*_, *θ*_*W*_, and Tajima’s *D* for all SNPs in the *D. yakuba* and *D. simulans* genomes, removing sites with missing or ambiguous data as well as heterozygous sites using libsequence [94]. We calculate *θ*_*π*_, *θ*_*W*_, and Tajima’s *D*3 for 5 kb windows moving in a 500 bp slide across the genome. For each window, we divide estimates by the number of sites per window with a minimum Illumina coverage depth of 20 reads, maximum coverage of 250 reads, MQ ≥ 20, consistent with the threshold used to identify SNPs, in order to estimate *θ*_*π*_ and *θ*_*W*_ per site. We compare *θ*_*π*_ per site for regions surrounding derived, segregating tandem duplications with regions surrounding derived, segregating, putatively neutral intronic SNPs from 8–30 bp of short introns, excluding windows with less than 4000 bp out 5000 bp that could be assayed for SNPs. We exclude second SNPs in a single 5 kb window, and exclude SNPs and duplicates that are found in the centromeric regions, which have unusually low diversity (Figures B-K in S1 File). We scaled diversity estimates by chromosome mean and standard deviation to produce a unit normal distribution and allow data to be combined across chromosomes. We then compared diversity at the 5000 bp window immediately surrounding SNPs to diversity for the window immediately surrounding duplications and the X chromosome using a single tailed Wilcoxon rank sum test (Table C in S1 File). Chromosome 2 in *D. yakuba* houses multiple segregating inversions [53, 54], which can cause atypical evolutionary dynamics and abnormal signals of diversity. Thus, we assayed data for chromosomes 2, 3 and X separately in *D. yakuba*. For *D. simulans*, data was combined across all autosomes, and the X chromosome in *D. simulans*. We plotted lowess smoothed regressions of diversity using a smoothing factor of $\frac{1}{10}$ from 50 kb upstream to 50kb downstream of a mutant (Figs 2–3). Tests of nucleotide diversity and plots of diversity surrounding duplicates and SNPs exclude a cluster of multiple duplications from 8.45 Mb-8.55 Mb which has abnormally low diversity (Figure I in S1 File).

## Supporting Information

S1 File Supporting Information. (PDF)

S2 File Contains Supplementary Text A-J. Text A: chromosome names vs number identifiers for *D. simulans*. Text B: DAVID gene ontology results for *D. simulans*. Text C: list of duplicated genes by chromosome for *D. simulans*. Text D: DAVID gene ontology results for *D. yakuba*. Text E: list of duplicated genes by chromosome for *D. yakuba*. Text F: chromosome names vs number identifiers for *D. yakuba*. Text G: list of fly stocks. Text H: Readme file. Text I: List of duplications for *D. simulans*. Text J: List of duplications for *D. yakuba*.
(ZIP)
