# Supplementary material for: Tandem Duplications and the Limits of Natural Selection in Drosophila yakuba and Drosophila simulans
Source: PLoS One. 2015 Jul 15;10(7):e0132184. doi: 10.1371/journal.pone.0132184 (PMC4503668; doi:10.1371/journal.pone.0132184)
Supplement: S1 File — (PDF) [file pone.0132184.s001.pdf]

Supporting Information

Table A. Wilcoxon Rank Sum Tests of Site Frequency Spectra

Species	Type	Type	W	Adjusted P-value
D. yakuba	Autosomal SNPs	Autosomal Duplicates	212	3.507×10^{-6}**
	X-linked SNPs	X-linked Duplicates	211	4.781×10^{-4}**
	Autosomal Duplicates	X-linked Duplicates	172	0.0128*
D. simulans	Autosomal SNPs	Autosomal Duplicates	268	2.981×10^{-6}**
	X-linked SNPs	X-linked Duplicates	113	0.2897
	Autosomal Duplicates	X-linked Duplicates	183.5	0.1848

* $P < 0.05$, ** $P < 0.01$

SNPs are derived from 8-30 bp of short first introns $\leq$ 100bp.

Table B. Kolmogorov-Smirnov Tests of Site Frequency Spectra

Species	Type	Type	D	Adjusted P-value
D. yakuba	Autosomal SNPs	Autosomal Duplicates	0.9333	3.868×10^{-7}**
	X-linked SNPs	X-linked Duplicates	0.800	5.235×10^{-5}**
	Autosomal Duplicates	X-linked Duplicates	0.5333	0.02625*
D. simulans	Autosomal SNPs	Autosomal Duplicates	0.8824	4.808×10^{-7}**
	X-linked SNPs	X-linked Duplicates	0.2941	0.4654
	Autosomal Duplicates	X-linked Duplicates	0.3529	0.2402

* $P < 0.05$, ** $P < 0.01$

SNPs are derived from 8-30 bp of short first introns $\leq$ 100bp.

Table C. Reduced Diversity Surrounding Tandem Duplications

Species	Chrom	Duplicate Diversity†	SNP Diversity†	W	single tailed P
D. yakuba	3	0.15	0.36	170168	0.006654
	2	0.49	0.36	115370.5	0.00567
	X	0.49	0.11	18078.5	0.5128
D. simulans	Autosomes	0.04	0.36	627683.5	2.267×10^{-7}
	X	0.12	0.32	13450.5	0.01819

†Mean Diversity estimates scaled by chromosome mean and variance to produce a unit normal distribution

Table D. EASE scores for gene ontology categories overrepresented in both *D. yakuba* and *D. simulans*

Functional Category	*D. yakuba*	*D. simulans*	*D. yakuba* Control	*D. simulans* Control
Chitin metabolism or cuticle	2.00	0.97	0.3098	0.1885
Immune defense to bacteria	1.44	1.59	0.8210	0.6985
Drug and toxin metabolism	1.37	2.32	< 0.02	< 0.02
Chemosensation	1.12	1.37	0.6597	0.2127

Table E. Estimated Number of Segregating Duplications on X and major Autosomes

Statistic	*D. yakuba*	*D. simulans*
Genome wide θ_W for tandem duplications	383	264
$E[S]$	5700	3800
σ_S	497	344
$E[S] + 2\sigma_S$	6800	4500
Genomic Coverage	13.4%	9.7%
Chao $E[S]$	3850	3940
Chao $E[S] + 2\sigma_S$	4190	4360
$E[S] + 2\sigma$, $N = 10^8$	8550	5700

Table F. Number of duplications necessary to cover segments of the genome

Percent Covered	Lower Bound (95% CI)	Upper Bound (95%CI)
5%	2,358	2,660
10%	4,912	5,360
25%	13,668	14,410
50%	33,191	34,427
90%	119,799	113,767

Table G. Multiply duplicated genes ($\geq$ 4 duplications)

Species	Gene	Number
D. yakuba	GE10684-PA	4
	GE13282-PA	4
	GE18810-PA	4
	GE18813-PA	4
	GE18814-PA	4
	GE20773-PA	4
	GE20774-PA	4
	GE25839-PA	4
	GE18811-PA	6
	GE18812-PA	6
D. simulans	CG2174-PD	4
	CG33466-PA	4
	CG4250-PA	4
	CG42566-PA	4
	CG33162-PA	15
	CG32022-PA	16
	CG5939-PA	27
	CG6533-PA	29
	CG6511-PA	32
	CG6517-PA	32
	CG6519-PA	32
	CG6524-PA	32

Table H. Mutation limited evolution in *D. yakuba* and *D. simulans* under large census size.

Species		Intron SNPs	Whole Gene	Recruit	Chimera
D. yakuba	μ	5.8×10^{-9}	1.17×10^{-9}	3.46×10^{-10}	3.70×10^{-10}
	θ_π	0.0138	0.00277	0.00082	0.00088
	T_e, $s = 0.01$, $100N_e$	74	360	1,220	1,140
	T_e, $s = 0.20$, $100N_e$	4	18	61	57

Species		Intron SNPs	Whole Gene	Recruit	Chimera
D. simulans	μ	5.8×10^{-9}	6.03×10^{-10}	2.42×10^{-10}	8.52×10^{-11}
	θ_π	0.0280	0.00291	0.00117	0.00041
	T_e, $s = 0.01$, $100N_e$	36	344	857	2430
	T_e, $s = 0.20$, $100N_e$	2	17	43	121

P_{sgv} from Hermisson and Pennings (2005) estimates the likelihood of adaptation from standing genetic variation under an additive model assuming neutral variation.
T_e (Gillespie 1991 and Maynard-Smith 1971) estimates the average time until establishment of a selective sweep from a new mutation in generations given that a site is under strong selection with beneficial mutation rate equal to θ_π. Estimates provide a lower bound on T_e.

Table I. Sojourn time and nearly neutral selection coefficient vs. population size

N_e	$2ln(2N_e)$	$s > \frac{1}{4N_e}$†	θ_{Dups}‡
10^8	38	2.5×10^{-9}	4.68×10^{-1}
10^6	30	2.5×10^{-7}	4.68×10^{-3}
10^4	20	2.5×10^{-5}	4.68×10^{-5}
10^2	11	2.5×10^{-3}	4.68×10^{-7}

† Time to loss given loss

‡ $4N_e\mu$ Given $\mu_{Dups} = 1.17 \times 10^{-9}$ from *D. yakuba*. s

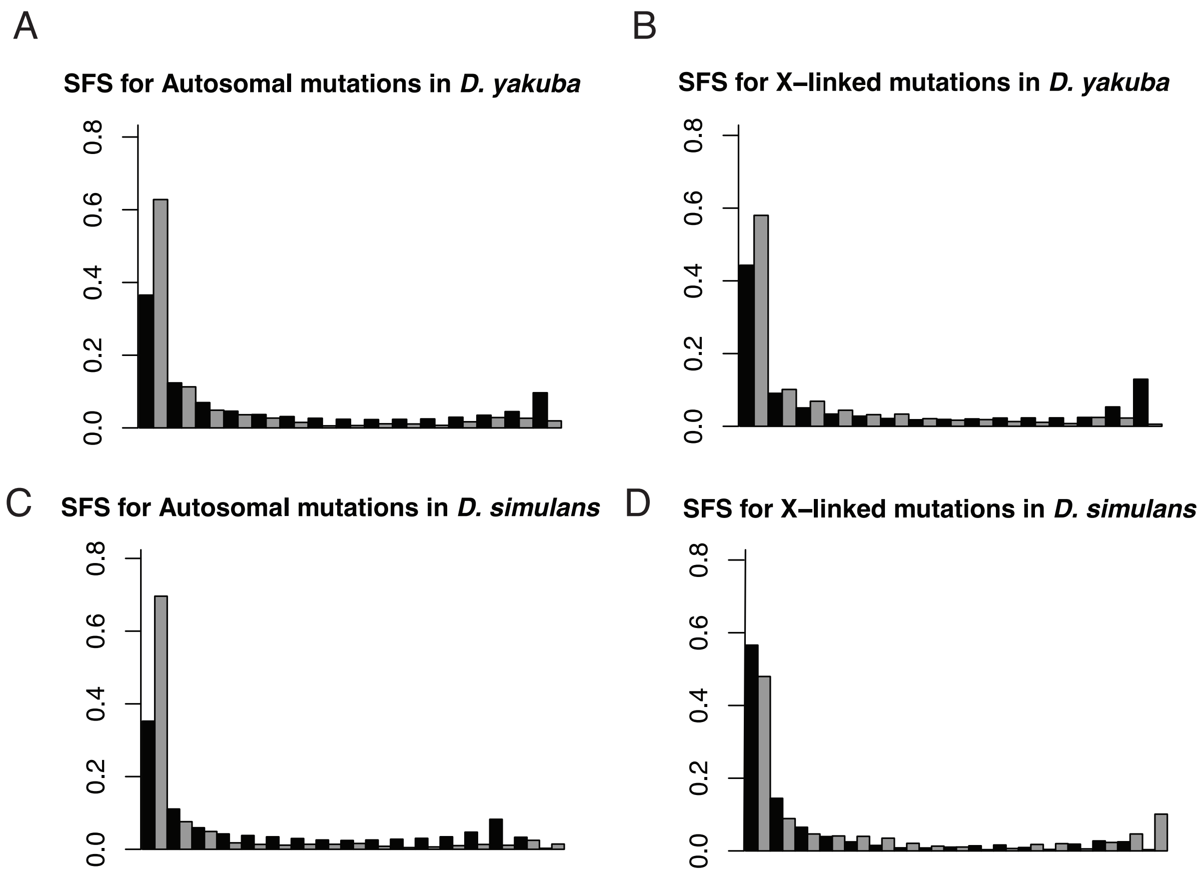

Figure A. Site frequency spectra for SNPs (black) and tandem duplications (grey) on the A) X and B) autosomes in *D. yakuba* and on the C) X and D) autosomes in *D. simulans.* Tandem duplications in *D. yakuba* and on the *D. simulans* autosomes show an excess of low frequency variants, consistent with detrimental phenotypic effects. The *D. simulans* X shows an excess of high frequency variants, consistent with widespread selection favoring duplicates on the X.

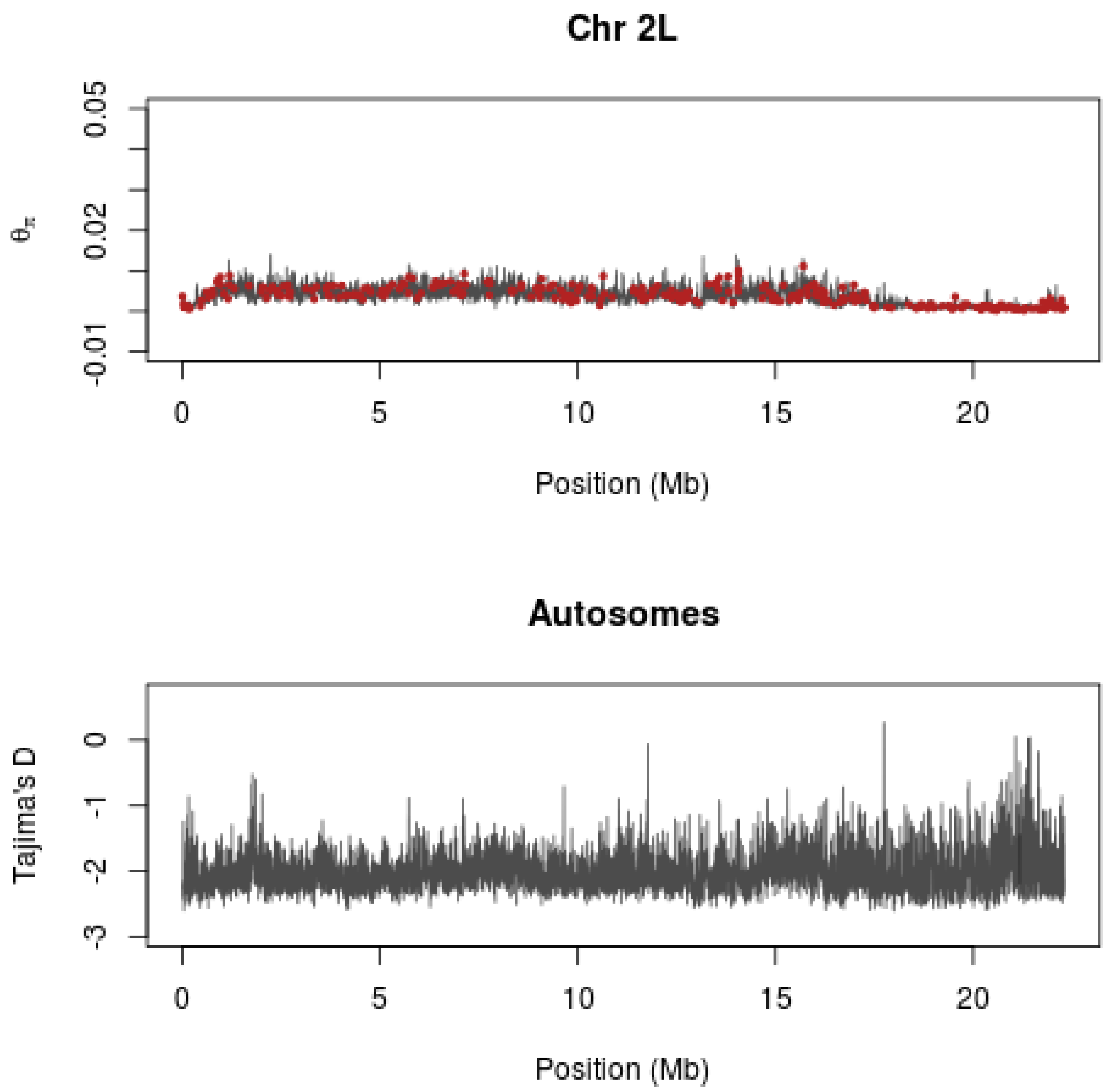

Figure B. Nucleotide Diversity θ_π and Tajima's D chromosome 2L in *D. yakuba* for 5kb windows with a 500 bp slide, showing only windows with 1 kb or more of sequence with coverage sufficient to call SNPS. Tajima's D is negatively skewed, consistent with recent population expansion and mean diversity θ_π is low surrounding centromeric regions. Locations and diversity values for duplications are marked in red.

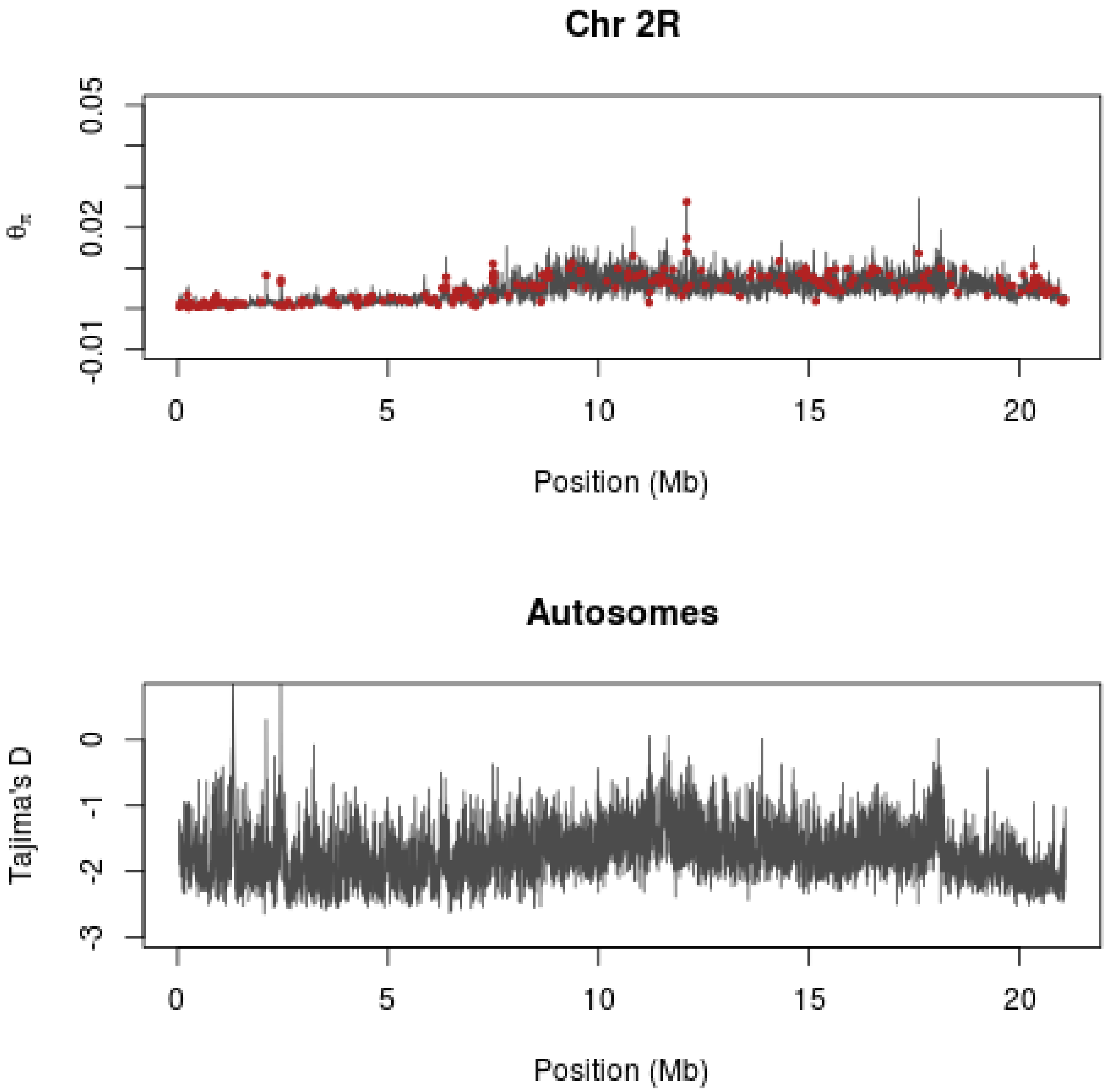

Figure C. Nucleotide Diversity θ_π and Tajima's D chromosome 2R in *D. yakuba* for 5kb windows with a 500 bp slide, showing only windows with 1 kb or more of sequence with coverage sufficient to call SNPS. Tajima's D is negatively skewed, consistent with recent population expansion and mean diversity θ_π is low surrounding centromeric regions. Locations and diversity values for duplications are marked in red.

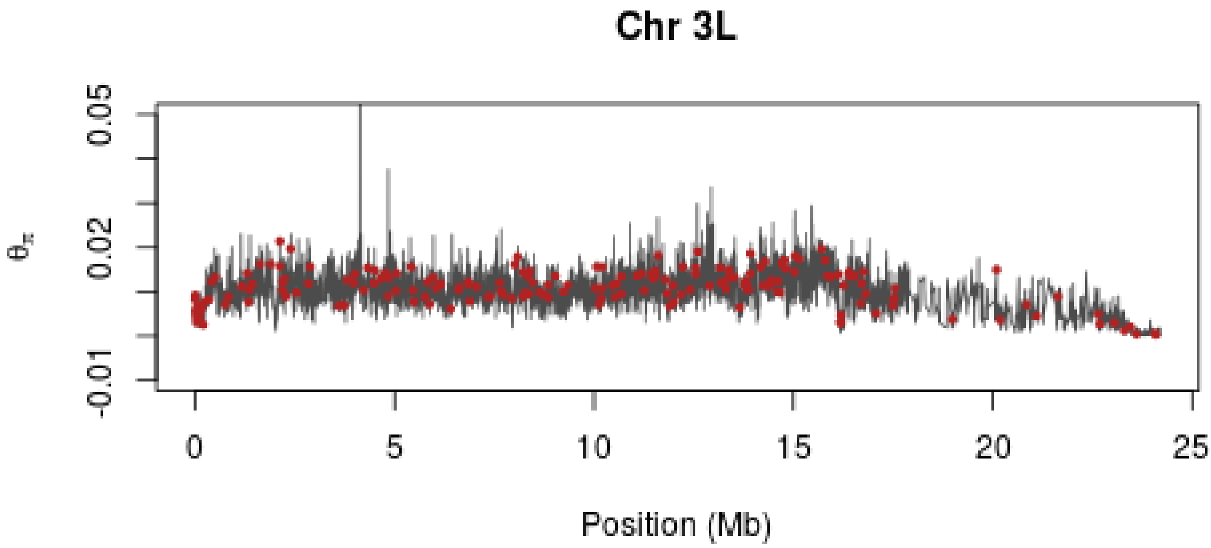

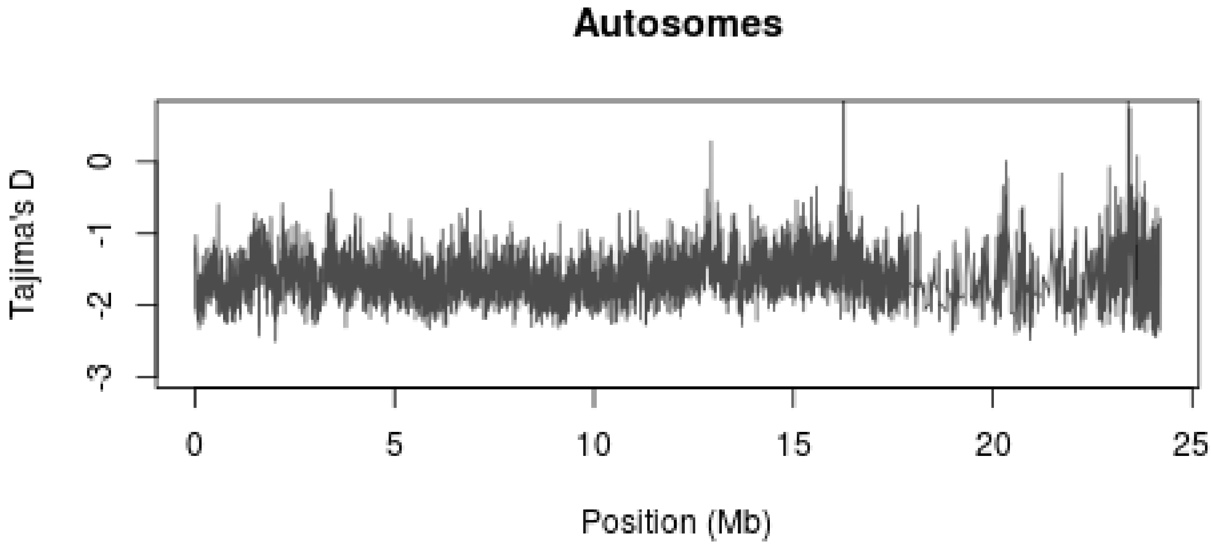

Figure D. Nucleotide Diversity θ_π and Tajima's D chromosome 3L in *D. yakuba* for 5kb windows with a 500 bp slide, showing only windows with 1 kb or more of sequence with coverage sufficient to call SNPS. Tajima's D is negatively skewed, consistent with recent population expansion and mean diversity θ_π is low surrounding centromeric regions. Locations and diversity values for duplications are marked in red.

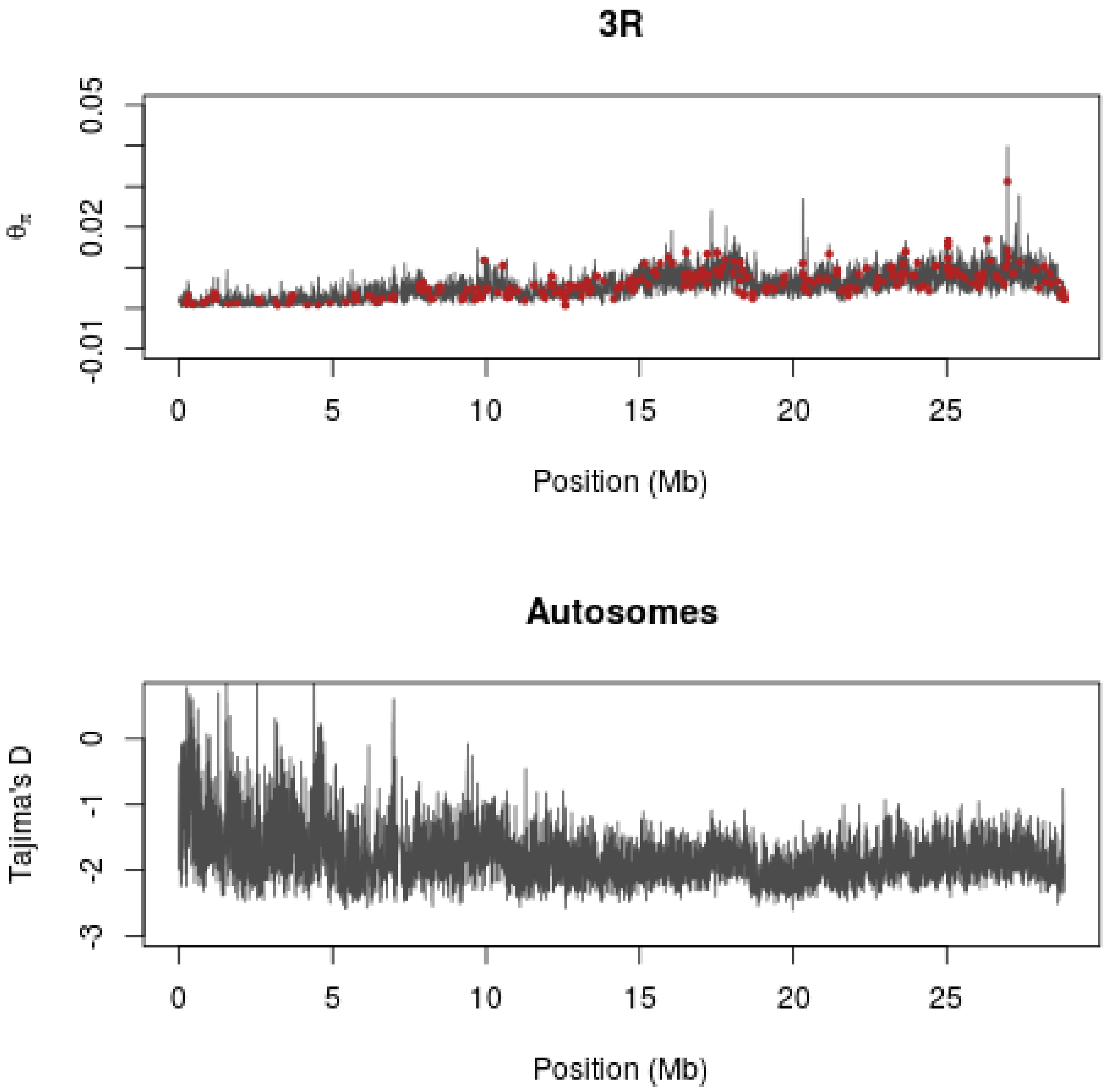

Figure E. Nucleotide Diversity θ_π and Tajima's D chromosome 3R in *D. yakuba* for 5kb windows with a 500 bp slide, showing only windows with 1 kb or more of sequence with coverage sufficient to call SNPS. Tajima's D is negatively skewed, consistent with recent population expansion and mean diversity θ_π is low surrounding centromeric regions. Locations and diversity values for duplications are marked in red.

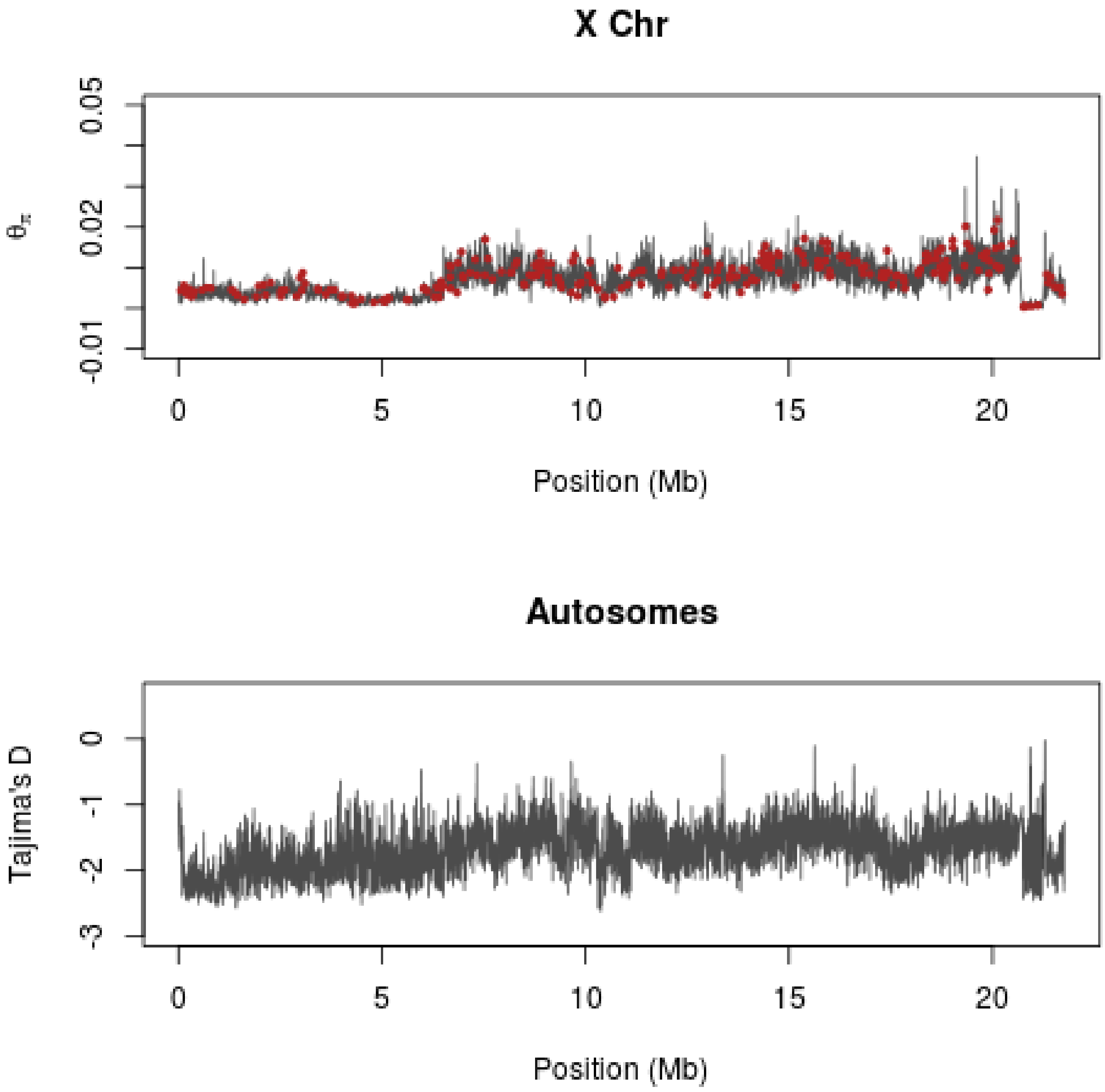

Figure F. Nucleotide Diversity θ_π and Tajima's D for the X chromosome in *D. yakuba* for 5kb windows with a 500 bp slide, showing only windows with 1 kb or more of sequence with coverage sufficient to call SNPS. Tajima's D is negatively skewed, consistent with recent population expansion and mean diversity θ_π is low surrounding centromeric regions. Locations and diversity values for duplications are marked in red.

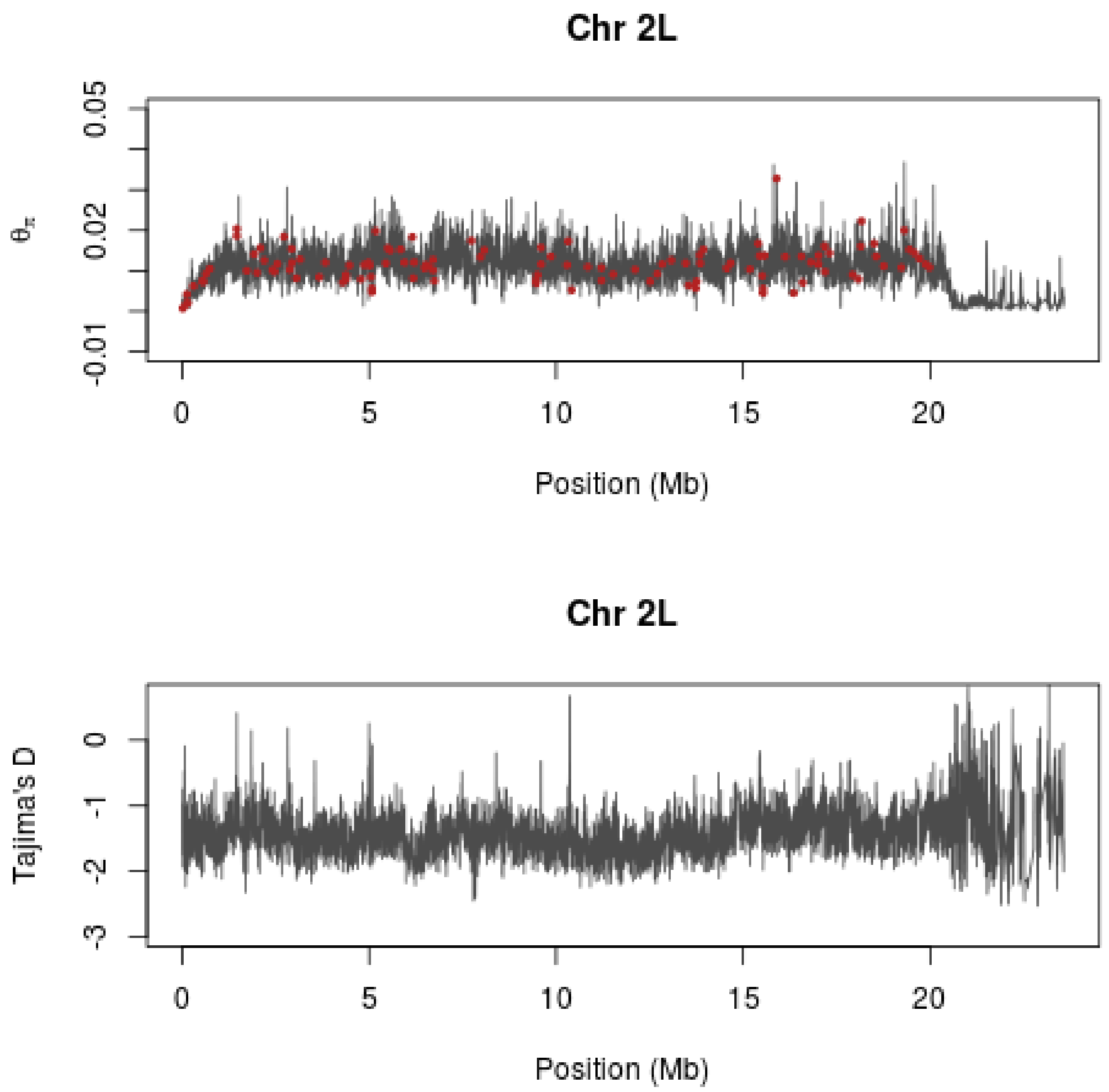

Figure G. Nucleotide Diversity θ_π and Tajima's D chromosome 2L in *D. simulans* for 5kb windows with a 500 bp slide, showing only windows with 1 kb or more of sequence with coverage sufficient to call SNPS. Tajima's D is negatively skewed, consistent with recent population expansion and mean diversity θ_π is low surrounding centromeric regions. Locations and diversity values for duplications are marked in red.

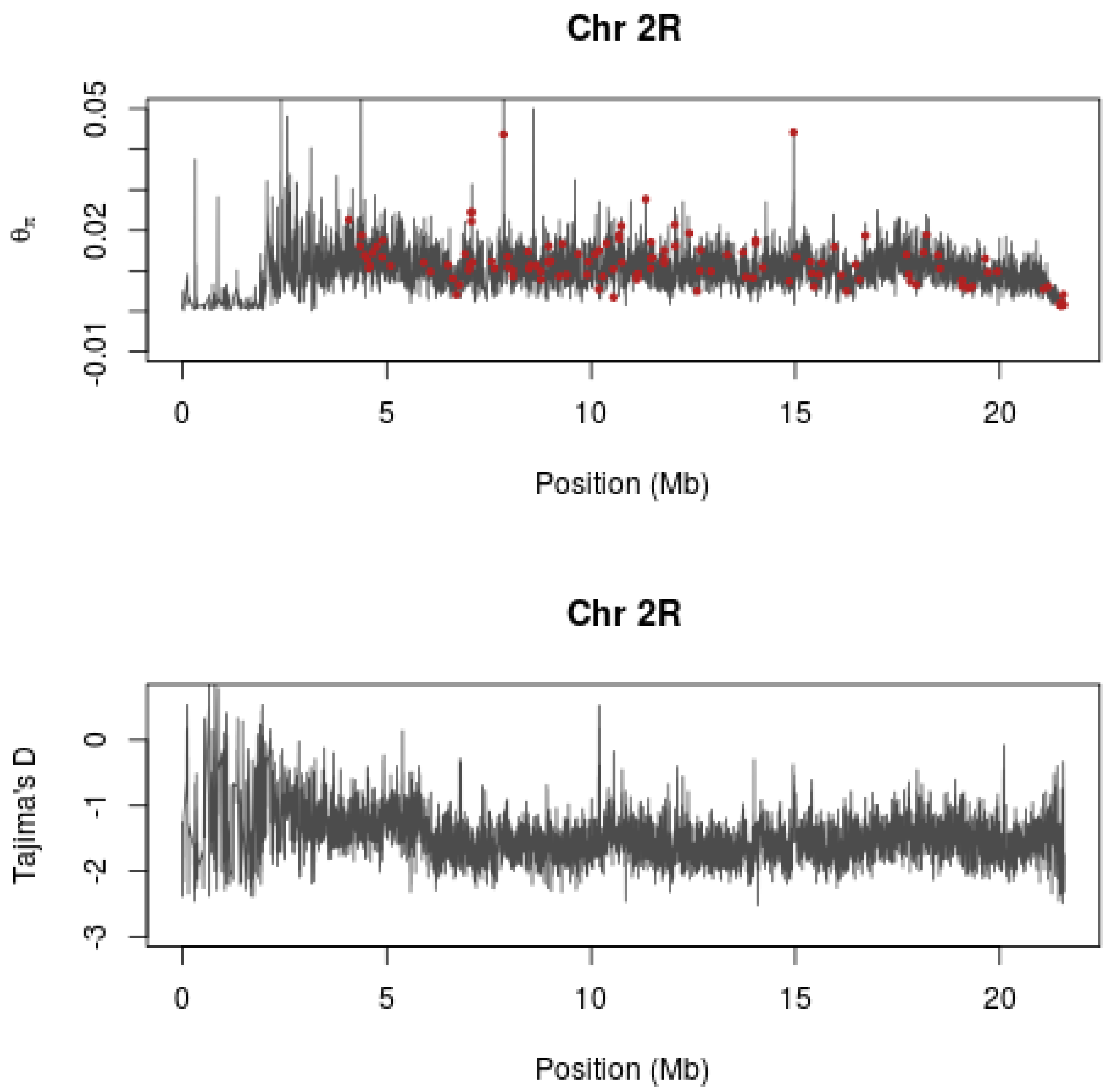

Figure H. Nucleotide Diversity θ_π and Tajima's D chromosome 2R in *D. simulans* for 5kb windows with a 500 bp slide, showing only windows with 1 kb or more of sequence with coverage sufficient to call SNPS. Tajima's D is negatively skewed, consistent with recent population expansion and mean diversity θ_π is low surrounding centromeric regions. Locations and diversity values for duplications are marked in red.

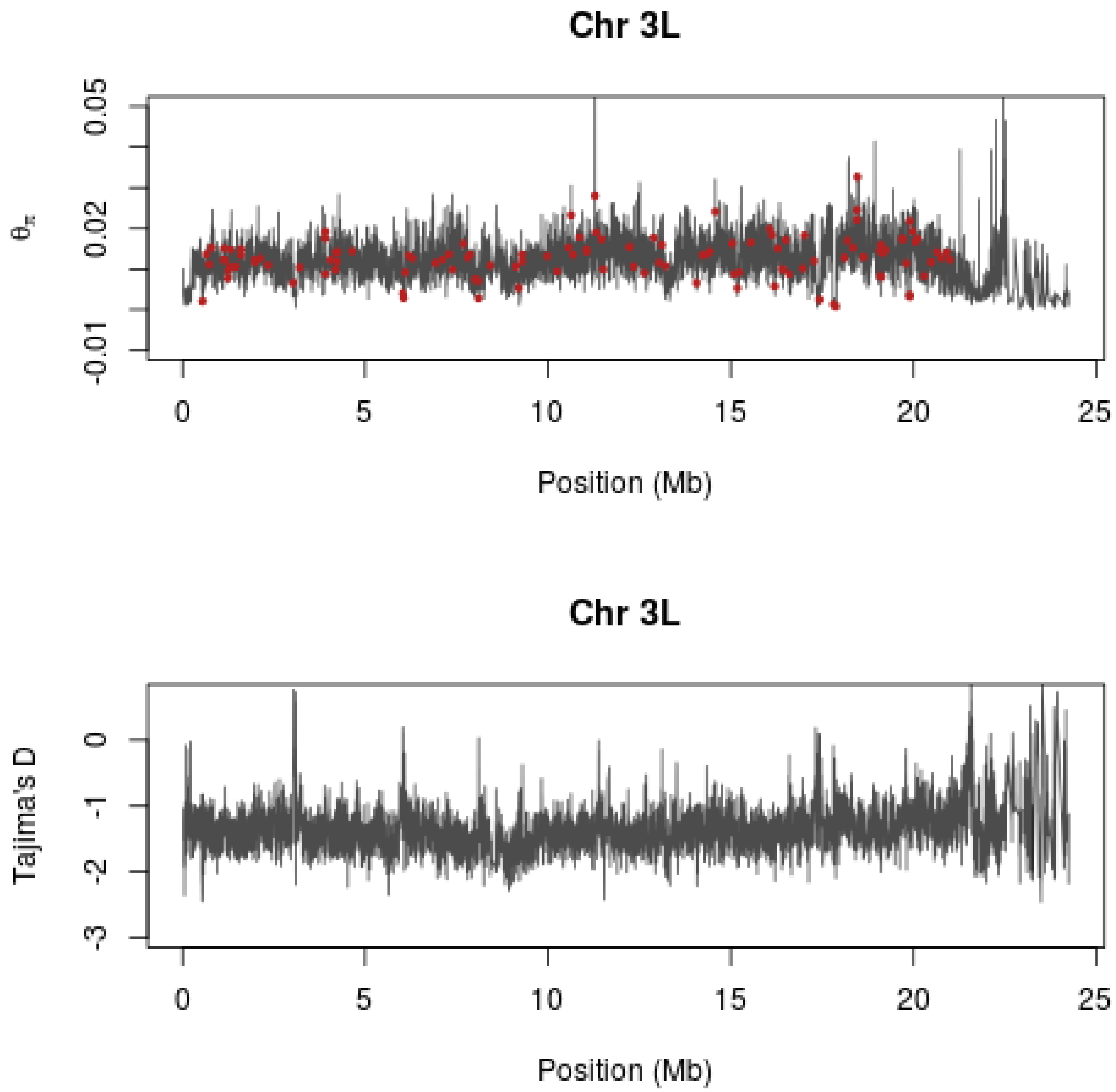

Figure I. Nucleotide Diversity θ_π and Tajima's D chromosome 3L in *D. simulans* for 5kb windows with a 500 bp slide, showing only windows with 1 kb or more of sequence with coverage sufficient to call SNPS. Tajima's D is negatively skewed, consistent with recent population expansion and mean diversity θ_π is low surrounding centromeric regions. Locations and diversity values for duplications are marked in red. Chromosome 3L is strongly affected by a cluster of duplications over 30 independent duplications of a genomic segment at roughly 8.5Mb which shows greatly reduced diversity.

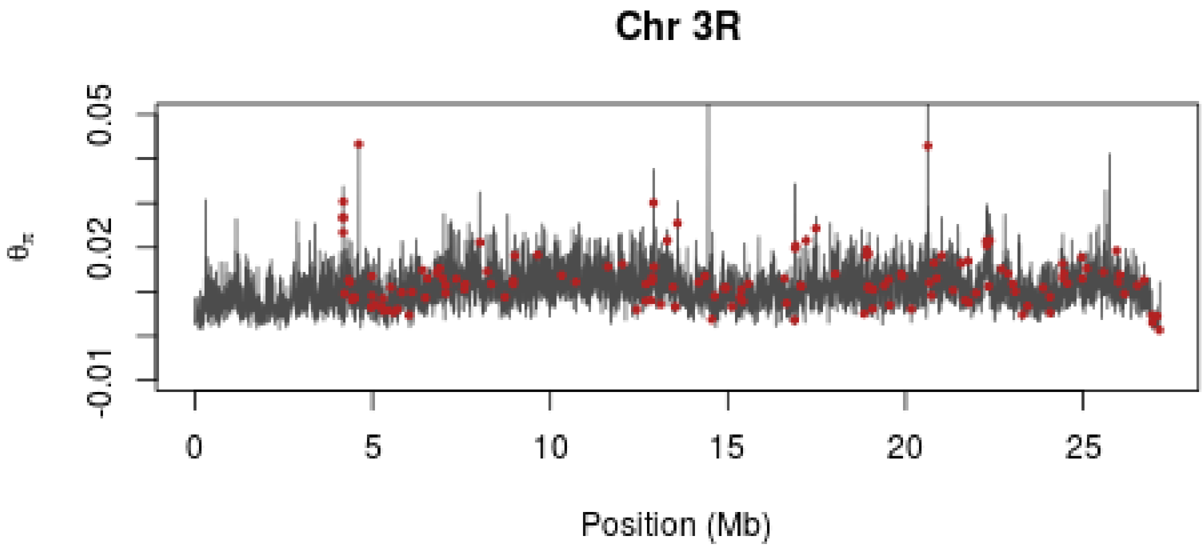

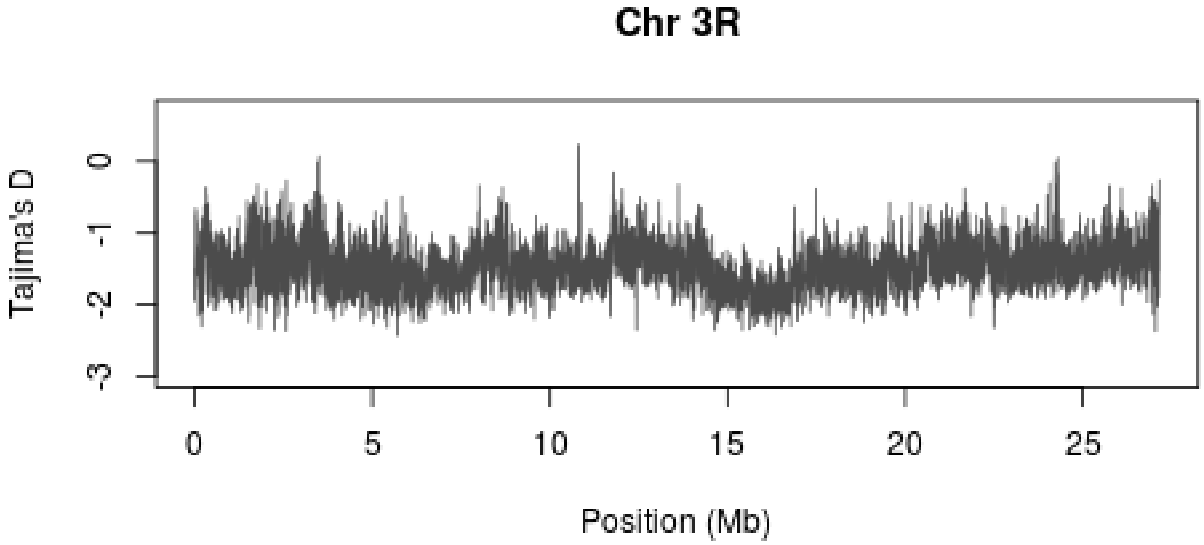

Figure J. Nucleotide Diversity θ_π and Tajima's D chromosome 3R in *D. simulans* for 5kb windows with a 500 bp slide, showing only windows with 1 kb or more of sequence with coverage sufficient to call SNPS. Tajima's D is negatively skewed, consistent with recent population expansion and mean diversity θ_π is low surrounding centromeric regions. Locations and diversity values for duplications are marked in red.

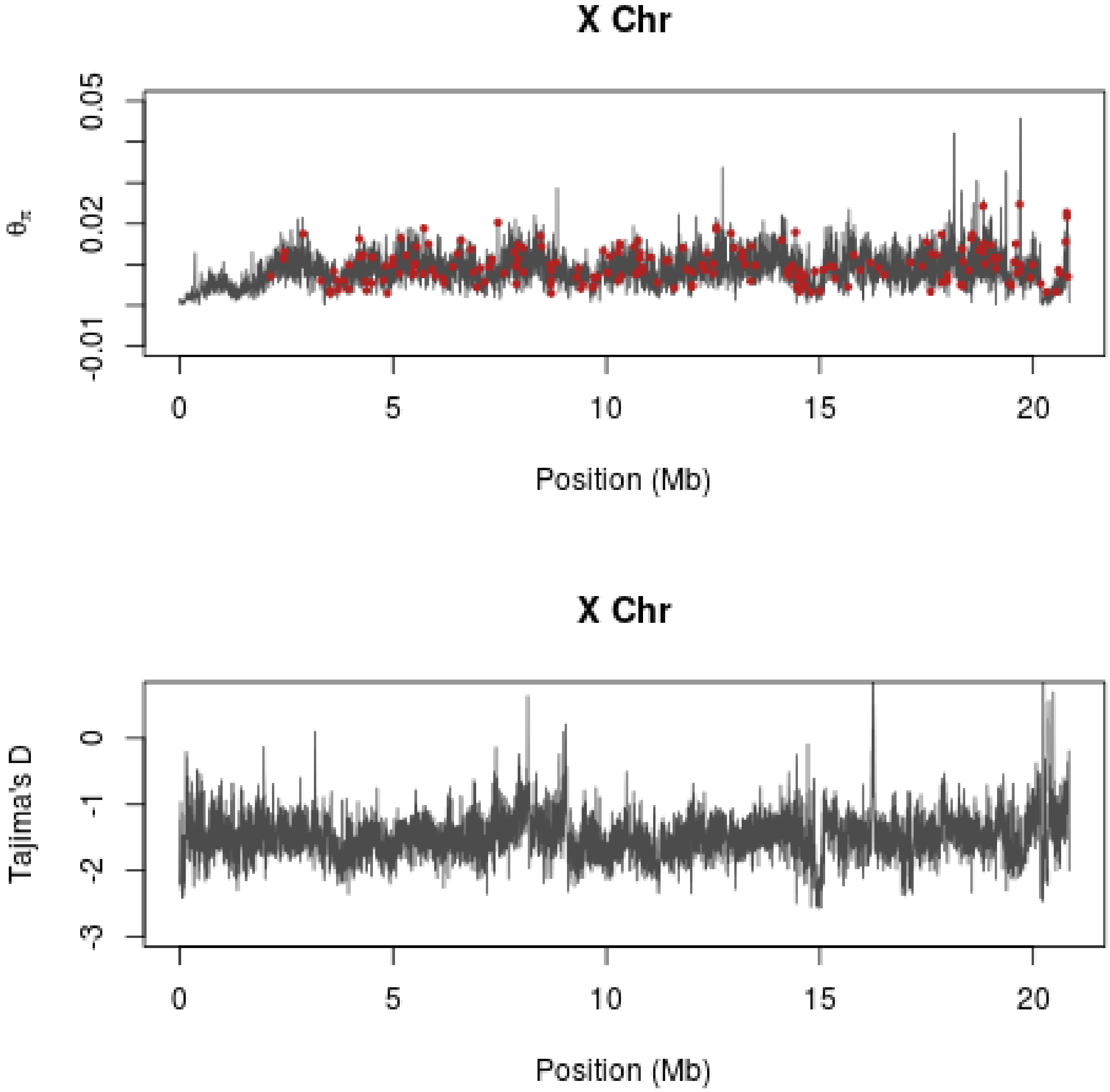

Figure K. Nucleotide Diversity θ_π and Tajima's D for the X chromosome in *D. simulans* for 5kb windows with a 500 bp slide, showing only windows with 1 kb or more of sequence with coverage sufficient to call SNPS. Tajima's D is negatively skewed, consistent with recent population expansion and mean diversity θ_π is low surrounding centromeric regions. Locations and diversity values for duplications are marked in red.

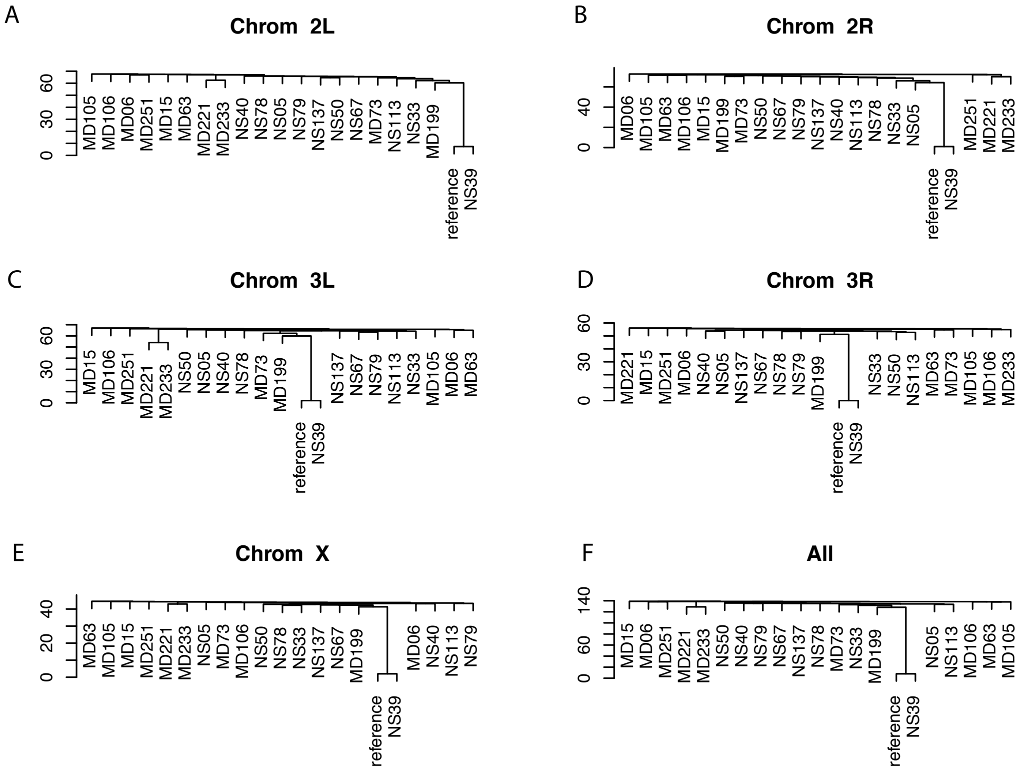

Figure L. Hierarchical clustering of intronic SNP data for *D. simulans* shows little population structure.

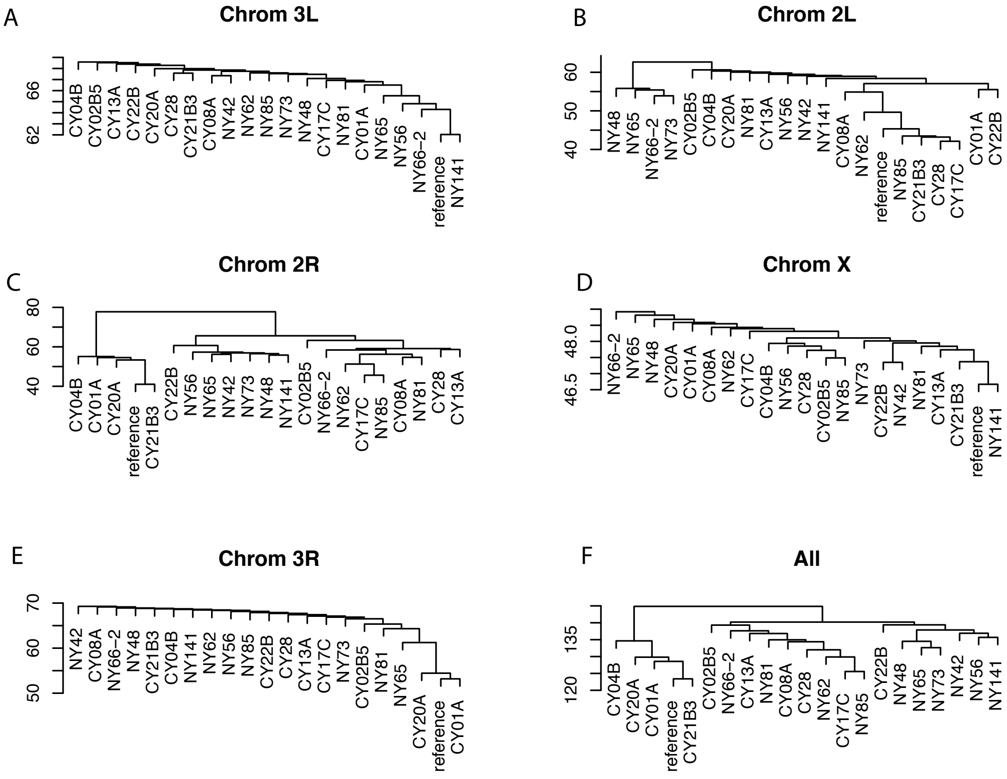

Figure M. Hierarchical clustering of intronic SNP data for *D. yakuba* shows population structure on chromosome 2L (B) and 2R (C), consistent with known inversions segregating on chromosome 2. Samples do not cluster strictly with respect to geography (A,D-E), indicating widespread gene flow between geographic locations and a single admixed population.
